# Toward an integrated framework of corporate venturing for organizational ambidexterity as a dynamic capability

**DOI:** 10.1007/s11301-021-00223-y

**Published:** 2021-06-05

**Authors:** Lysander Weiss, Dominik K. Kanbach

**Affiliations:** 1 Dr. Ing. h.c F. Porsche AG Chair of Strategic Management and Digital Entrepreneurship, HHL Leipzig Graduate School of Management, Jahnallee 59, 04109 Leipzig, Germany; 2 HHL Leipzig Graduate School of Management, Jahnallee 59, 04109 Leipzig, Germany

**Keywords:** Organizational ambidexterity, Corporate venturing, Corporate innovation, Dynamic capabilities, Strategic management, Corporate entrepreneurship, M1, M10, M13

## Abstract

In times of changing business environments, firms must constantly renew their competitive advantage by establishing dynamic capabilities. While often attempting to employ this in corporate venturing activities, they face the challenge of simultaneously exploring new and exploiting existing business opportunities. Examining possible approaches to mastering this feat of ‘organizational ambidexterity’ reveals an extensive but scattered picture. To better integrate this effort by assessing how corporate venturing is linked with organizational ambidexterity in the literature and identifying possible organizational setups, this systematic literature review builds on a sample of 172 studies. Based on different dimensions of dynamic capabilities, the analysis indicates that corporate venturing may take a solely explorative or an exploitative role, or balance both, to directly enable organizational ambidexterity, following a ‘trade-off’, respectively ‘paradox’, school of thought. As a result, this paper identifies four different setups of corporate venturing in an integrated framework, based on the ability and approach to enabling organizational ambidexterity. Here, the synthesis in the proposed framework of the studies examined allows differentiating between not directly ambidextrous separated or integrated corporate venturing and directly ambidextrous contextual or interlinked corporate venturing. As a novel contribution to the fields of strategic management, organizational change and corporate entrepreneurship, this integrated perspective suggests an often overlooked, potentially more strategic role for corporate venturing in the strategic renewal of a firm’s competitive advantage, thus building the basis for further empirical research on strategic corporate venturing approaches for organizational ambidexterity and their application in practice.

**Supplementary Information:**

The online version contains supplementary material available at 10.1007/s11301-021-00223-y.

## Introduction

The recent COVID-19 crisis and broader trends of rapid technological development, globalisation and climate change underline the need for many established companies to renew their competitive advantages. Due to these continuous changes, the world in which today’s businesses operate has become not only riskier but also more volatile, uncertain, complex and ambiguous (Schoemaker et al. 2018). In the past, such dynamic environments characterised specific fast-moving industries, for example, the high-tech sector. Today, however, this describes the ‘new normal’ for most companies across numerous industries (Barreto 2010). In this context, faith in a definite strategic plan is fading and adapting to rapid changes constitutes a major strategic challenge that many organisations face (Du and Chen 2018).

To overcome this challenge and create and capture value from arising opportunities for sustainable competitive advantage, firms increasingly turn to higher-order capabilities that can handle any environment and cope with insufficient insight, foresight and broad understanding (Schoemaker et al. 2018). Extending the resource-based view in Barney’s (1991) work leads to describing these as unique and difficult to replicate ‘dynamic capabilities’ (DCs) (Ambrosini and Bowman 2009; Teece 2007; Teece and Pisano 1994). Consequently, for more than two decades, an established research stream has examined these organizational and strategic processes and routines ‘to integrate, build, and reconfigure internal and external competencies to address rapidly changing environments’ (Teece et al. 1997, p. 516). Using this logic, larger firms are not helplessly lost in the storm but, indeed, can undergo transformation and strategic renewal by utilising their great pool of available resources (Majumdar 2000; Schmitt et al. 2018; Simsek and Heavey 2011). As Covin and Miles (1999) pointed out, strategic renewal is an ambiguous term used for various phenomena. Through the lens of strategic management taken here, it describes the pursuit of new competitive advantages for the firm, which is required in a dynamic environment and may be achieved through various entrepreneurial activities. This approach contrasts with the view of strategic renewal as a specific activity within strategic entrepreneurship, dealing expressly with the adoption of a new strategy (Hill and Georgoulas 2016; Reimsbach and Hauschild 2012; Simsek and Heavey 2011).

Such strategic renewal presents a significant challenge because established companies already have an existing—typically successful—business to run. Thus, they cannot afford to focus only on renewing themselves but, instead, must explore future viable business opportunities while exploiting their existing competitive advantage (March 1991). As a result, established firms increasingly find themselves in tense situations that require doing what they do well while determining what they will do well in the future (Ireland and Webb 2007). Extensive research, driven primarily by the pivotal works of Tushman and O’Reilly (1996) clarifies that companies that manage this tension by simultaneously exploring new and exploiting existing business opportunities with ‘organizational ambidexterity’ (OA) can succeed in a changing world through greater innovation, better financial performance and overall higher survival rates (O’Reilly and Tushman 2011, 2013). Thus, it can be concluded that, to deal with the ambidexterity challenge and successfully develop and renew their competitive advantages in a dynamic environment, established companies must recombine and integrate new and existing resources, constituting a dynamic capability (Hueske and Guenther 2015; O’Reilly and Tushman 2013; Snehvrat et al. 2018; Tushman and O'Reilly 1996).

However, an ongoing debate in the context of both theory and practice considers precisely how a firm can establish such dynamic capability of organizational ambidexterity (Turner et al. 2013). Here, frequently examined approaches often lie in in the encompassing concept of ‘corporate entrepreneurship’, describing the use of innovative entrepreneurial activities in a corporate context to pursue the strategic renewal of competitive advantage, often differentiated into the specific forms of ‘strategic entrepreneurship’ and ‘corporate venturing’ (Hill and Georgoulas 2016; Kuratko and Audretsch 2013; Kuratko et al. 2015; Narayanan et al. 2009; Phan et al. 2009). As can be seen in this study and existing literature, the interplay of these differentiated but interconnected forms, with strategic entrepreneurship that may result in new business, and corporate venturing (CV) that may result in the strategic renewal of the firms’ competitive advantage, possibly leads to ‘strategic corporate venturing’, in which simultaneous opportunity-seeking and advantage-seeking behaviour can be found in new business creation processes (Bierwerth et al. 2015; Hill and Georgoulas 2016; Narayanan et al. 2009; Raisch and Tushman 2016; Reimsbach and Hauschild 2012; Simsek and Heavey 2011). Thus, with such ‘strategic corporate venturing’, established companies may develop dynamic capabilities in their management practices, and therefore entrepreneurial activities for continuous innovation and strategic renewal, enabling them to create and advance new combinations of resources through simultaneous exploration and exploitation to address the ambidexterity challenge (Corbett et al. 2013; Eisenhardt and Martin 2000; Hill and Georgoulas 2016; Ireland and Webb 2007; Madsen 2010).

While the overall relevance and connection of CV to organizational ambidexterity is evident in the existing literature, exactly how it may work as a dynamic capability to solve the ambidexterity challenge and thus contribute to the strategic renewal of a firm’s competitive advantage is the subject of substantial but fragmented and ongoing discussion in the fields of strategic management (e.g. Schoemaker et al. 2018), organizational science (e.g. Birkinshaw et al. 2016), and corporate entrepreneurship (e.g. Kuratko et al. 2015; Shankar and Shepherd 2019). Even though various conceptual and empirical studies examine how CV might contribute to OA (e.g. Hill and Birkinshaw 2014), a shared understanding and an overview of the possible roles for CV in regard to OA are still missing (Hill and Birkinshaw 2006; Narayanan et al. 2009; Rossi et al. 2019a). Scholars often argue that CV and other business functions mostly perform either a solely explorative or solely exploitative task (Gutmann 2019). However, recent research suggests a directly ambidextrous role for CV, which potentially would lead to strategic corporate venturing as a dynamic capability in the continuous renewal of the firm (Blindenbach-Driessen and Ende 2014; Hill and Birkinshaw 2014; Holotiuk and Beimborn 2019). Therefore, the ongoing debate provides different standpoints regarding the approaches and strategic roles of CV for OA, which leads to a certain confusion not only in theory but also in practice.

The latter is particularly relevant at the moment, as a renewed interest in corporate venturing is observable at the corporate level, a presence that some even call a new golden age of CV (Battistini et al. 2013; Kanbach and Stubner 2016). In contrast to previous waves of corporate venturing that usually ended in cycles of recession, this golden age already has persisted for a comparably long time and, thus, shows some signs of maturity (Birkinshaw et al. 2002; Birkinshaw and Hill 2005, Burgelman and Välikangas 2005; Weiblen and Chesbrough 2015). Here, established CV modes, such as corporate venture capital or internal new venture development, are now complemented by newer approaches, such as (digital) innovation labs, corporate accelerators or corporate incubators (Gutmann 2019; Kanbach and Stubner 2016; Shankar and Shepherd 2019). Often being part of the overall trend towards more ‘open innovation’, some of these modes seem to better bridge the gap between the start-up and the corporate world and may, therefore, potentially enable simultaneous exploration and exploitation (Hill and Georgoulas 2016; Schroll and Mild 2012; Weiblen and Chesbrough 2015). However, firms continue to struggle with the successful employment of such new and old CV modes for long-term growth and corporate renewal purposes, especially due to uncertainty about operational links with the overall strategy in terms of integration and separation of the corporate venturing and other business functions (Dushnitsky and Birkinshaw 2014; Narayanan et al. 2009).

This presents a research opportunity to link CV more closely with contemporary strategic management concepts and organizational theory to fully capture its non-financial benefits, which may be especially relevant in the described new CV modes, and could demonstrate how companies can leverage CV to their strategic advantage (Dushnitsky and Birkinshaw 2014; Hill and Georgoulas 2016; Narayanan et al. 2009; Shankar and Shepherd 2019). To examine this in the context of the described challenge for established firms to adapt their competitive advantage to changing environments through dynamic capabilities, we can therefore formulate the following research question: *“How is corporate venturing linked with organizational ambidexterity in the literature, and which different setups can be identified accordingly?”.*

Such an examination contributes to the corporate entrepreneurship domain and the fields of organisation and strategic management research, in which the necessary managerial processes and structures for OA as a DC often remain vague and the question of how to potentially overcome the ambidexterity challenge with corporate venturing for the creation of future competitive advantage offers a compelling avenue for investigation (Hill and Georgoulas 2016; Narayanan et al. 2009; O’Reilly and Tushman 2013). To examine and conceptualise the possible approaches of CV for OA as a DC, the present study builds on the methodology of a systematic literature review to describe the intersection of the different concepts, thus possibly extending the current theory across them. As a result, we follow an inductive approach that focuses on the concepts’ interfaces which are not defined beforehand but developed as a result of the explorative collection and analysis of relevant publications (Aguinis et al. 2018; Fisch and Block 2018; Kraus et al. 2020; Webster and Watson 2002). Although most systematic literature reviews in the domain of (corporate) entrepreneurship cover broad topics, we thereby follow the call of Kraus et al. (2020) to deal with topics in an in-depth manner, focusing on CV as a specific approach for OA. This way we can eventually provide a new integrated view of corporate venturing, organizational ambidexterity and dynamic capability concepts. Hereby, the proposed integrated framework clearly shows the possibility of ambidextrous corporate venturing that can contribute to the strategic renewal of an established firm’s competitive advantage by contextually supporting ambidextrous individual behaviours or providing interlinked structures and processes. This allows to differentiate ambidextrous forms of CV that follow a paradoxical view of ambidexterity from structurally separated CV with a trade-off view.

With these new insights for organizational setups of (strategic) CV, and the proposed conceptual basis for further empirical research into possible roles of CV for the strategic renewal of firm’s competitive advantage, we aim to provide both scholars and practitioners with a clearer view and a new, sound perspective on the potential role of CV in the strategic management of a firm as a relevant contribution (Hill and Georgoulas 2016; Kraus et al. 2020).

## Methodology and sample

The chosen method, a systematic literature review, allows us to seek answers to the research question on the intersection of different theoretical concepts. We hereby adopt the position that such pure literature reviews can focus on patterns and connections among various empirical findings in a broader scope, thus playing a role in post-hoc theorizing previous empirical results and research (Frank and Hatak 2014). This choice is therefore valid in the present context, with a substantial but scattered and still-emerging body of research across the fields of DC, OA and CV, which needs conceptual integration as a possible foundation for further research and evidence-based management.

**Fig. 1:**
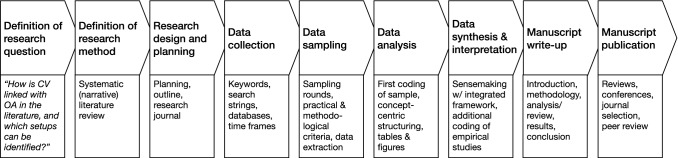
Research process

To ensure objectivity and reproducibility in the systematic literature review, the research design follows the structured approach of Tranfield et al. (2003), which became a quasi-standard for systematic literature reviews in the last decade (Breslin et al. 2020). Accordingly, the examination follows a structured process (see Fig. 1), taking the overall structure of an empirical article with the introduction (1) describing the background and motivation to the research the topic, as well as the central question and the contribution to be expected; the methodology (2) explaining the research design in a transparent, reproducible way; the analysis (3) examining key concepts and their relationships; the results (4) providing a conceptual model of an integrated framework to provide answers to the research question; and the conclusion (5) discussing the findings within the context of the broader theories, as well as the boundaries and implications for future research and practice (Fisch and Block 2018).

Further detailing this process with a transparent description of the research design, including the data collection and sampling procedure and specifications of the analytical method applied, should increase the credibility and reproducibility of the analysis and results (Aguinis et al. 2018). The *systematic data collection* to identify relevant publications was based on carefully selected relevant keywords for the research question and context. In an a priori overview of the topic, these were derived from literature reviews and seminal articles in the topics of CV, OA and DC, which also ensured consistency of the terms within the overall debate (Frank and Hatak 2014). From the given keywords, titles and definitions included in the a priori overview, we could build a list of possible keywords (see Appendix I), including synonyms and specifications, as different terms often classify comparable phenomena in entrepreneurship research (Kraus et al. 2020). This choice allowed us to design specific search strings, primarily focusing on the intersections of DC, OA and CV through the use of the boolean AND, while including the possible specific keywords as synonyms with boolean OR (see Appendix II).

The keywords and search strings were discussed with experts from both theory and practice and crosschecked in educational and grey literature (Kraus et al. 2020). Consequently, we could minimise the typical trial and error process to find the right balance of depth and breadth in the search by adjusting the available keywords in only two rounds (Frank and Hatak 2014). The subsequent search was conducted in March 2020 on the EBSCOHost database, while new articles were added until March 2021. EBSCOHost was used for practical (as access to the database was given) and theoretical reasons as it is a recommended database, particularly in entrepreneurship research (Frank and Hatak 2014; Kraus et al. 2020). To avoid limiting our search to one database, we also conducted a crosscheck on the Google Scholar database in privacy mode to ensure reproducibility, as discussed in Gusenbauer and Haddaway (2020). While Google Scholar is often viewed negatively by academics, examinations from recent years recognise it as a valid database, covering sources in social sciences and humanities especially well (Harzing and Alakangas 2016). Additionally, another crosscheck has been conducted on the Scopus database before the publication of the article.

**Fig. 2:**
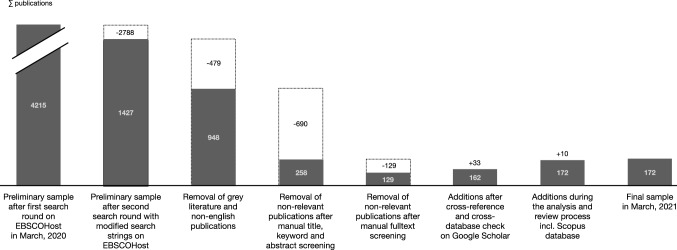
Sampling process

As a basis for the following *data sampling process* (see Fig. 2), a first round was conducted with the complete search strings in titles, keywords and abstracts, including most of the specific keywords as possible synonyms (see Appendix II). This search led to a first sample of 4,215 articles, which was found to be too broad in an initial screening, covering many articles that were too specific for one of the topics concerning our aim of reviewing the overall academic debate specifically for the intersection of DC, OA and CV. A second search round, now focusing only on the most prominent keywords, led to a more relevant, reduced sample of 1,427 publications (see Appendix II). In the next step, non-academic sources and grey literature were excluded from this sample as recommended by Kraus et al. (2020), and only English-language articles were included, which reduced the selection further to 948 articles.

We did not apply further methodological criteria, such as limiting the search to specific journal ranks. We also included academic conference articles and book chapters to capture newer, emerging views in the academic debate on the intersection of the topics as recommended by Frank and Hatak (2014). Further, we did not limit the time frame of the sample to cover both earlier and more current discussions within the given topics. Consequently, the remaining 948 publications were screened for duplicates as well as manually for their relevance to the research question by reading first through titles and keywords (if given), as well as abstracts if a decision could not be taken before, leading to a preliminary sample of 258 articles (Kraus et al. 2020). Hereby, the manual screening focused explicitly on sources with examinations of structures, processes and behaviours at the intersection of CV, OA and DC in established companies, i.e. excluding articles examining other aspects such as business model innovation (e.g. Ricciardi et al. 2016), new product development (e.g. Katila and Ahuja 2002), technological innovation (e.g. Ahuja et al. 2008), physical spaces (e.g. Moultrie and Lewis 2005) or taking different perspectives such as learning (e.g. Zollo 2009). This reduction was followed by another round of manual screening following an identical focus for the remaining articles, reviewing the entire corpus of articles for those still in question, leading to a relevant sample of 129 articles.

Within the identified articles in this sample, the reference list of most relevant papers and literature reviews were used to identify possibly missing publications, complemented by a forward citation check on Google Scholar to track where these publications were cited afterwards (Webster and Watson 2002). In contrast to the previous database search focused only on the interconnections of the different theoretical concepts, this manual step allowed for a broader search scope and purposefully included the addition of seminal papers for each specific concept to provide a good foundation for the analysis. Additionally, another crosscheck with the final search strings on Google Scholar was used to compare results (sorted by relevancy) until saturation was reached with no relevant new concepts and only few new sources added to the sample. These crosschecks resulted in another 33 publications with mostly seminal papers from the manual cross-reference checks added, increasing the total sample to 162 publications. In the course of further analysis and reviews of the manuscript and a final crosscheck on the Scopus database before publication, ten more publications, especially recent ones, were added, making up a final sample of 172 articles (see Fig. 2). These multiple sampling steps should ensure a relevant and current sample with both seminal papers on each concept to examine, as well as specifically on their interconnections.

Discussing the final sample with fellow researchers and in conference submissions confirmed that it provided a comprehensible basis for the desired purpose of building an integrated framework by reviewing the connection and possible setups of CV for OA through the lens of DC in the following *systematic review and analysis*. Here, we follow a concept-centric approach to evaluate the underlying concepts of CV for OA, specifically regarding their interconnections, to address the stated research question in the context of DC (see Appendix III). This focus ensures the critical balance of breadth and depth, as it allowed us to limit the scope to seminal articles of the three main concepts, complemented by the identified publications focusing on DC-OA, DC-CV and OA-CV interconnections (Fisch and Block 2018). Accordingly, every publication in the sample was coded thoroughly based on its contribution to the background and interconnections of OA, DC and CV to conduct the systematic review and analysis (see Appendix IV). Subsequently, different organizational setups of CV along the identified DC and OA logics could be coded, resulting in the integrated framework of CV for OA as a DC (see Appendix V).

Based on the iteratively and inductively developed coding scheme (see Appendix III), the systematic review and analysis provide the theoretical basis for the subsequent integration of relevant empirical CV studies within the dimensions of the resulting integrated framework (see Appendix V,VI). Thus, this systematic literature review goes well beyond a summary of the relevant scientific evidence, aiming to extend the existing theories of CV, OA and DC on their interfaces, and thus following the call of Rauch et al. (2014) for a better synthesis and interpretation of empirical findings in the domain of entrepreneurship. Consequently, the following analysis and synthesis of the examined literature focus on concepts, not authors, in a nonchronological manner, to derive an integrated conceptual framework as a novel contribution, accompanied by explanatory figures that complement the given supplementary material to further ensure the right balance of breadth and depth (Fisch and Block 2018; Kraus et al. 2020; Webster and Watson 2002).

## Analysis: systematic review

### Dynamic capabilities for organizational ambidexterity and corporate venturing

The review and analysis of the identified literature (see Appendix IV) demonstrate that the concept of dynamic capabilities provides a suitable theoretical foundation for organizational ambidexterity and corporate venturing, as these entrepreneurial activities aim to build and renew the competitive advantage of firms, are rooted and embedded in high-performance routines and processes and operate inside the firm (Teece and Pisano 1994).

Extending the resource-based view, DCs can explain why (and how) companies can achieve a competitive advantage independent of valuable, rare, inimitable and non-substitutable resources that tend to lose their competitive edge in (at least moderately) dynamic or uncertain environments (Teece 2007, 2018). By this logic, DCs may become a sustainable competitive advantage in themselves as a higher-order capacity of the organisation to continuously create, extend and modify its resource base, including all tangible, intangible and human assets and resources (Helfat and Peteraf 2009). Because DCs often remain vague, the concept can serve as an overarching theory for different organizational routines and processes by which firms achieve new resource configurations to adapt to changing markets over time, examples of which appear throughout the literature (e.g. Eisenhardt and Martin 2000; Vogel and Güttel 2013). This particularly enables the identification of exploring or exploiting routines and processes as potential DCs in CV through a systematic literature review, thereby answering the call from Helfat et al. (2007) to specify the particular DCs under investigation, as different types of DCs may perform different tasks. Even more specifically, capabilities may describe not only abilities but also processes or routines, organizational learning and managerial decision-making, all of which may enable CV units to explore and/or exploit new and existing opportunities (Barreto 2010). Thus, the lens of DC should enable us to integrate different CV processes for OA into a standard view.

Relatedly, to apply this theoretical lens it can be helpful to categorise and distinguish the distinct forms of dynamic capabilities along one or more suitable dimensions, which generally enhances the understanding of how a study fits in the broader nature of dynamic capabilities. Hereby, Schilke et al. (2018) call specifically for the further integration of different DC dimensions and typologies to provide coherence within the concept. We therefore decided to follow this call in our analysis, finding the *types of processes* as in Teece et al. (1997) or Teece (2007), the *functional domains* as in Eisenhardt and Martin (2000)*, the hierarchies of capabilities* as in Collis (1994) and the *focal unit of analysis* as in Adner and Helfat (2003) to be the most suitable dimensions, as they were widely used in the literature examined for this study:

*Functional domains* describe various organizational processes and functions in which DCs can manifest, such as acquisitions, alliances, product innovation or research and development (Anand et al. 2010; Easterby-Smith et al. 2009; Eisenhardt and Martin 2000). More generally, DCs can be present in functions that represent change routines (e.g. product development), strategic analysis (e.g. investment) and especially creative managerial (thus, entrepreneurial) acts (e.g. new markets, new business development) (Helfat and Peteraf 2009; Katkalo et al. 2010). As a common denominator, relevant functional domains should provide the possibility for knowledge acquisition and sharing, the continuous change of operating processes and resources and interaction with the external environment for new assets, moderated by decision-making assessments (Easterby-Smith et al. 2009). All of these may arise in specific CV functions, or more generally, in the organizational ability to simultaneously explore and exploit, as the following analysis confirms.

*Different types* of DCs build in particular on the work of Teece (2007), who divides the DC microfoundations for analytical purposes into sensing, seizing and transforming processes as an elaboration from coordination/integrating, learning and reconfiguring processes proposed in the seminal works of Teece and Pisano (1994) and Teece et al. (1997). Here, *sensing* means detecting risks and chances before rivals; *seizing* includes the implementation and realisation of identified opportunities by innovating and implementing new systems, possibly by leveraging existing ones; and *transforming* (sometimes also called reconfiguring or renewing) describes the enhancing, combining, protecting and possible reconfiguring of the tangible and intangible assets of the organisation, with the appropriate resources, structures and capabilities to reshape itself and, perhaps, its ecosystem for future growth (Schoemaker et al. 2018; Teece 2007). These types have been used extensively in DC research, especially at the intersection with other concepts, i.e. intersecting with value creation and value capture concepts from Katkalo et al. (2010), as distinct but related to March’s (1991) dimensions of exploration (sensing) and exploitation (seizing), and as a theoretical lens for corporate venturing activities (e.g. Helfat and Winter 2011; Martin and Eisenhardt 2004; Teece 2007). While newer elaborations exist, such as sensing, organising, value capturing and renewing types in Teece et al. (2020) that may particularly help a better operationalisation in practice, the described differentiation in sensing, seizing and transforming is currently the most empirically and theoretically established one. Therefore, this typology may offer a relevant dimension to move towards a common view for exploring or exploiting activities that may occur in CV (O’Reilly and Tushman 2011; Schilke et al. 2018).

Furthermore, the dimension of *hierarchies* describes the processes and routines that usually influence other capabilities, leading to zero- or lower-level ordinary capabilities that operate in the present and higher-level dynamic capabilities for reshaping the future (Collis 1994; Helfat and Winter 2011; Schilke et al. 2018; Teece 2014). Consequently, the higher-level activities alter how the company makes a living (i.e. its ordinary capabilities) to maintain external fitness (Teece 2007, 2018; Winter 2003). As a result, DCs can build sustainable competitive advantage over time; however, that might need to include an update of the DCs themselves, suggesting even more levels of dynamic capabilities (Helfat and Winter 2011; Wang and Ahmed 2007; Winter 2003). However, independent of the exact number of hierarchies, higher-level DCs enable firms to identify promising configurations of existing, lower-level, ordinary competencies and assets; to renew, (re-)assemble and orchestrate them; and to exploit them within an innovative and agile organisation (Schoemaker et al. 2018). With this logic, examining whether CV and OA appear on the same or different hierarchies of (dynamic) capabilities for their further integration becomes crucial.

Finally, the dimensionalisation of DCs in different functions, types and hierarchies enables examining them in different *focal units of analysis*. Moving beyond the original organizational level, various studies have examined DCs on managerial/individual, team/unit, network, and extra-organizational levels, suggesting that integrating CV and OA as a DC might also require a multilevel view (Schilke et al. 2018).

**Fig. 3:**
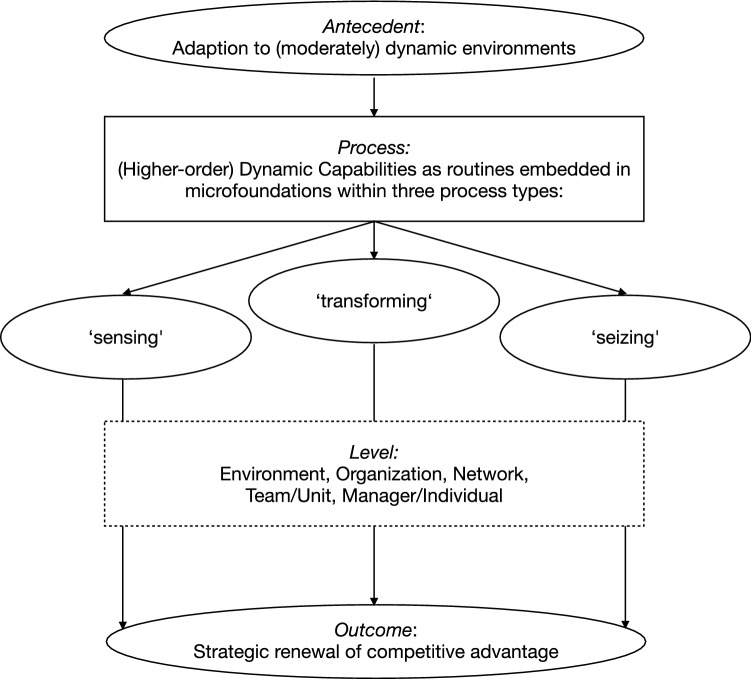
The DC view

All in all, we can conclude from the analysis that this multidimensional view of DC confirms its potential as an appropriate lens through which to examine the role of CV for OA, as corporate venturing can appear as a functional dimension that might be involved in sensing, seizing and transforming processes for exploring and/or exploiting as a higher- or lower-order (dynamic) capability, embedded in individual/managerial and unit/organizational routines (see Fig. 3). This confirms the proposition by Augier and Teece (2009), who stated that the DC framework might be a useful foundation for understanding the process of opportunity sensing and seizing, as well as strategic renewal, all of which CV may enable to tackle the OA challenge.

### Organizational ambidexterity as a dynamic capability

The OA challenge of simultaneously exploring and exploiting existing and future opportunities for sustainable competitive advantage, as March (1991) particularly has suggested, is now well documented in the literature (O’Reilly and Tushman 2008, 2011; Teece and Pisano 1994). Here, *exploring* is typically used to describe the recognition of input and generation, assessment, and further evaluation of new ideas, which requires flexibility and adjustment. *Exploiting* includes the absorption of new opportunities in a more established set of routines, with predictable behaviours that demand efficiency to gain traction and avoid mistakes (Madsen 2010). While these are usually seen as opposites, the ‘holy grail’ of OA lies in the organisation’s ability to leverage existing assets and capabilities from its mature business to gain competitive advantages in new areas (Eisenhardt et al. 2010; O’Reilly and Tushman 2013). Therefore, an examination of how exploration and exploitation activities play out in the entrepreneurial CV function may provide relevant approaches to successfully realising OA (see Appendix IV).

Further assessing organizational ambidexterity through the lens of dynamic capabilities, it is often viewed as either a DC or complementary to the DC perspective (Birkinshaw et al. 2016; Jansen et al. 2009; O’Reilly and Tushman 2011; Teece 2007). The last decade in particular produced many conceptual studies on the integration of both OA and DC perspectives, thus building the case to examine OA within the DC framework (Alänge and Steiber 2018; O’Reilly and Tushman 2013; Snehvrat et al. 2018). Overall, the literature agrees that dynamic capabilities are anchored in the organizational ambidexterity of the firm to help the organisation reconfigure its resource base to exploit existing competencies and explore new ones. This enables further integration of OA in the dimensions of DC as a basis for an integrated view (Taylor and Helfat 2009).

While the often-conceptual OA studies included in this review generally do not address specific *functional domains*, Gupta et al. (2006) described the interaction between exploration and exploitation having a positive impact on specific organizational functions, such as new product development, thus implying the possibility of finding or influencing OA in different functions, such as CV.

Looking more specifically at the different *types of processes*, some scholars have recently tried to integrate the dimensions of exploring and exploiting into the DC microfoundations of sensing, seizing and transforming (Birkinshaw et al. 2016; Madsen 2010; Popadiuk et al. 2018). Often, exploring and exploiting are seen as or related to sensing and seizing abilities to create and capture value (Katkalo et al. 2010; O’Reilly and Tushman 2013; Teece 2007). However, as the true challenge of OA lies not in exploring or exploiting alone but in the simultaneous pursuit of both, this view might be insufficient. Instead, the integration of the sensing and seizing processes to create new sustainable competitive advantages that could be classified as transforming must play a key role here (O’Reilly and Tushman 2008, 2013).

Interestingly, the view that transforming (understood as balancing exploration and exploitation) may be of greater importance than the capabilities of sensing and seizing appears in the *hierarchical dimensions* of OA as a DC. Several scholars describe OA as a critical DC (only) for its ability to strategically integrate or balance exploration and exploitation, suggesting that the pure existence of exploration and exploitation alone may not be sufficient to function as a DC (Benner and Tushman 2003; Katkalo et al. 2010; Raisch and Birkinshaw 2008). Consequently, Birkinshaw et al. (2016) concluded that sensing and seizing often arise in operating units, while transforming abilities are developed and implemented at a higher level to coordinate and balance sensing and seizing. Thus, sensing and seizing for exploring and exploiting could be classified as ordinary or lower-order dynamic capabilities, while balancing both for transforming could be a higher-order DC, proposing a multilevel approach to enabling OA to function as a DC.

This multilevel view, mirrored in the different *focal units of analysis* for OA in the literature, emerges from there. While the term ‘organizational ambidexterity’ originally referred to an organizational (or senior leadership), higher-order level, at which the transformation through balancing exploration and exploitation takes place, scholars increasingly have looked at other focal units of analysis, such as unit ambidexterity or individual ambidexterity, to address the reality of strategic management in multi-unit firms (Jansen et al. 2012; O’Reilly et al. 2009). In broader terms, the question of where OA can take place represents different schools of thought around the question of how exploring and exploiting are discrete (duality) or complementary (dualism). Hereby, the ‘trade-off view’ of duality follows March’s (1991) logic, arguing that exploration and exploitation are important for long-term success but fundamentally incompatible, as they compete for the same scarce resources (Almahendra and Ambos 2015; Gupta et al. 2006). Some scholars, such as Christensen (1997), originally accepted this notion, describing companies as either explorative or exploitative. However, in the context of OA, the more prevalent suggestion for successfully managing these discrete activities is built around separating them within the organisation, e.g., in different units or over time, but possibly integrating them on the corporate level through top management’s ambidextrous behaviour (O’Reilly and Tushman 2011; Raisch and Birkinshaw 2008; Simsek et al. 2009; Teece 2007). Even though this perspective is widely established in theory and practice, it remains open to criticism because the success of this solution heavily depends on top management’s presupposed abilities to understand and balance the different sensing and seizing activities, presenting a complex trade-off (Chen 2017; Lavie et al. 2010; Jansen 2011).

As an alternative, some studies have implied that stability and change may jointly contribute to organizational effectiveness. In that way, explorative and exploitative activities may be complementary, especially if required resources in a knowledge economy are not scarce, but (externally) available and could support both (Farjoun 2010; Gupta et al. 2006; Marín-Idárraga et al. 2016; Zollo and Winter 2002). In this ‘paradox view’ of dualism, the distinction between exploration and exploitation becomes a matter of degree on a continuum rather than two discrete poles (Gibson and Birkinshaw 2004; Lavie et al. 2010; Papachroni et al. 2014). For instance, individuals engaged in ordinary activities may also exercise some degree of experimentation, and others engaged in creative tasks will also use certain repeatable routines. In the same way, explorative units will still build stable structures and controls, while exploitative ones may embrace some degree of variation. Bringing both together, organisations may use knowledge transfer and sharing, cross-training, interlinked processes or networks to foster simultaneous exploration and exploitation (Farjoun 2010; Stadler et al. 2014). This view consequently leads to alternative organizational approaches, allowing business units and other individuals in addition to senior managers to participate in both exploration and exploitation simultaneously, giving the DC of transforming back into the organisation (Birkinshaw et al. 2016; Raisch et al. 2009; Schuh et al. 2017). Consequently, in this case, the DC of OA lies not in the separate organizational structure and its integration by top management, but in the processes in which units and individuals interact, such as cross-functional interfaces, social networks and the context that allows individuals and teams to make their own judgements within a given strategic frame (Agostini et al. 2016; Gibson and Birkinshaw 2004; Jansen et al. 2009). However, Callegar and Rai (2021) found this pursuit of simultaneous exploration and exploitation within and across business units to be the most complex configuration.

**Fig. 4:**
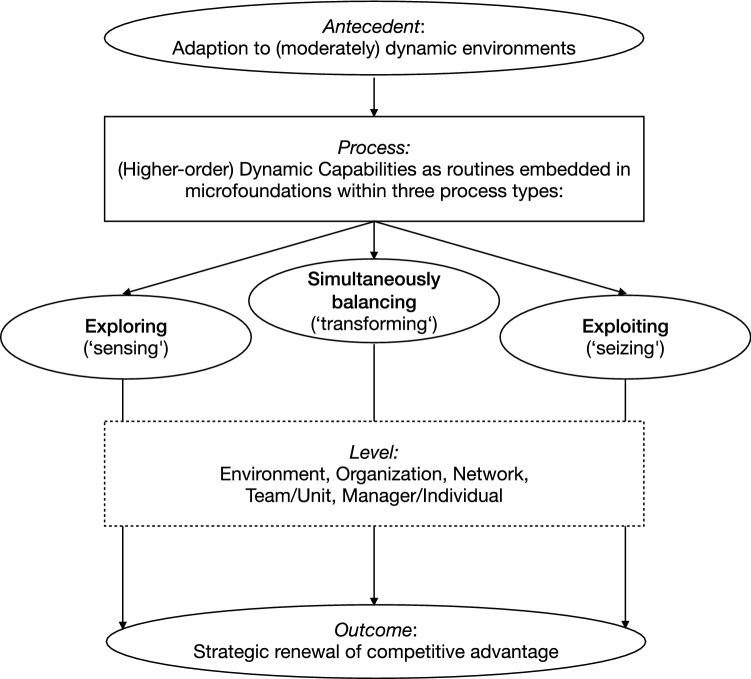
The DC view of OA

Thus, integrating OA in the different focal units of analysis that the DC framework proposes leads to a conclusion that the balance of exploration and exploitation can present itself at different levels of an organisation, which may adopt structural ‘trade-offs’ or contextual and processual ‘paradox’ approaches (see Fig. 4). As a clear integration of these different approaches currently lacks in the literature, combining these different views within an integrated framework may contribute to a clearer multilevel view of OA as a DC, in which CV could contribute to either lower-level sensing or seizing in the trade-off view or higher-level transforming in the paradox view (Agostini et al. 2016; Birkinshaw et al. 2016; O’Reilly and Tushman 2013).

### Corporate venturing as a dynamic capability

Examining the application of CV through the lens of DC, the literature indicates the need for established firms to find and exploit new opportunities by extending their domain of competence through new resource combinations that corporate entrepreneurship can achieve (Burgelman 1984b; Kuratko and Audretsch 2013). Consequently, research in the twenty-first century links corporate entrepreneurship, in the sense of innovation with the use of entrepreneurial activities in a corporate context, to the creation of sustainable competitive advantage as a source of profitable growth, indicating interconnectedness with OA and DC based on their sharing the same intended outcome (Corbett et al. 2013; Kuratko and Audretsch 2013). While many definitions of this concept exist, today it is mostly differentiated into ‘strategic entrepreneurship’ and ‘corporate venturing’, which can take place at various levels of the organisation (Hill and Georgoulas 2016; Kuratko et al. 2015; Narayanan et al. 2009; Phan et al. 2009).

Hereby, *strategic entrepreneurship* usually describes entrepreneurial activities at the intersection of strategy and entrepreneurship that involve the simultaneous balancing of opportunity- and advantage-seeking behaviour through organizationally consequential changes for building (sustainable) competitive advantage for the firm, thus addressing the organizational ambidexterity challenge on a strategic level as a dynamic capability (Covin and Miles 1999; Gutmann 2019; Hitt et al. 2011; Ireland et al. 2003; Ireland and Webb 2007; Kuratko and Audretsch 2013; Kuratko et al. 2015; Raish and Tushman 2016). As a key differentiator, strategic entrepreneurship normally involves strategy, organisation, product/market categories or business model changes in the existing enterprise—which may or may not involve new business creation (Hill and Georgoulas 2016; Simsek and Heavey 2011).

In contrast, *corporate venturing* involves creating (investing in/adding) a new business (i.e. products, services, business models) to the established organisation to pursue financial or strategic objectives (Covin and Miles 2007; Gutmann 2019; Hill and Georgoulas 2016; Kuratko and Audretsch 2013; Sharma and Chrisman 1999). While scholars often agree on this overall common theme of corporate venturing, the activity can manifest itself in various forms. On a general level, these are often divided into internal, external and cooperative types depending on where an opportunity comes from or is realised (Gutmann 2019; Kuratko and Audretsch 2013). Furthermore, various CV modes exist in the sense of specific configurations such as corporate venture capital, skunkworks, accelerators, open innovation programmes, incubators, intrapreneurship programmes, company builders, digital labs and many others, in which terms are often used ambiguously, reflecting an ever-growing variety of CV in practice (Dushnitsky and Birkinshaw 2014; Narayanan et al. 2009; Reimsbach and Hauschild 2012; Rossi et al. 2019b).

As a result, especially contemporary CV modes that often focus on collaboration with external start-ups in the sense of open innovation, or building internal start-ups, may play a novel role in the strategic renewal of a firm’s competitive advantage (Gutmann 2019; Rigtering and Behrens 2021; Shankar and Shepherd 2019). These different modes also reflect the varying levels and structures in which CV can appear in the organisation, which is sometimes differentiated as dispersed or focused CV, usually involving the possibility that CV is moderated by a specific CV unit or new venture division that may act as a link to the core business, corporate management and ecosystem and has the responsibility to create new business for the firm (Burgelman 1984a; Hill and Birkinshaw 2008; Leten and Van Dyck 2012; Reimsbach and Hauschild 2012; Zahra 1991).

Across these different forms of CV, many ambiguities remain, i.e. in terms of autonomy, relatedness, extent of innovation, nature of sponsorship or overall strategic importance (Covin and Miles 2007; Narayanan et al. 2009; Kuratko et al. 2014; Hill and Georgoulas 2016). Particularly for the coordinating CV units, (strategic) objectives are often not fully understood, partly due to the fact that access to internal units is often limited (in contrast to publicly available corporate venture capital functions with well-understood financial objectives), although this is currently changing with more of these units acting increasingly publicly (Gutmann 2019; Hill and Birkinshaw 2008; Kanbach and Stubner 2016; Reimsbach and Hauschild 2012; Shankar and Shepherd 2019).

Therefore, corporate venturing may or may not result in the strategic renewal of a firm’s competitive advantage, depending on the various configurations with different objectives and modes, and potentially moderated by a specific CV unit that manages the processes (Bierwerth et al. 2015; Burgelman 1984a; Hill and Georgoulas 2016; McGrath et al. 2006; Narayanan et al. 2009; Raisch and Tushman 2016; Reimsbach and Hauschild 2012). Consequently, the many different established and contemporary internal and external forms of CV must be considered in examining how CV is linked with OA, as long as their tasks relate to the creation of new business. Additionally, we must also consider the possibility that CV is moderated by a specific unit, which may act as a vehicle to deliver innovation to the parent company (Gutmann 2019; Rigtering and Behrens 2021).

In this context, we can conclude from the coanalysis (see Appendix IV) that CV could make a company more entrepreneurial and potentially act as a dynamic capability, as the integration of entrepreneurship in the organisation and strategy helps to build and renew the firm’s competitive advantages (Helfat and Winter 2011; Kuratko and Morris 2003; Martin and Eisenhardt 2004; Teece 2007). Indeed, the topic of integrating CV as an entrepreneurial process in the DC concept has garnered increasing interest in the past decade (Hill and Georgoulas 2016; Narayanan et al. 2009). However, as the existence of innovation alone would not make corporate venturing a relevant dynamic capability, if and how it is used to reconfigure the resource base of the company and to change its long-term position in existing or new markets, remains to be seen (Covin and Miles 1999; Kuratko and Audretsch 2013). In this sense, CV as a DC would need to not only create new businesses but particularly leverage the parent company’s resources to build competitive advantage against other established companies and start-ups, and therefore also influence the existing organisation in the sense of normally differentiated strategic entrepreneurship (Wolcott and Lippitz 2007). Therefore, CV as a DC is limited to modes that succeed in bringing both new business and change to the organisation in the form of ‘strategic corporate venturing’, suggesting that not every company fully benefits from the strategic potential of corporate venturing (Enkel and Sagmeister 2020; Shankar and Shepherd 2019; Williams and Lee 2009).

However, a successful case of strategic corporate venturing may be able to address the organizational ambidexterity challenge by exploring or exploiting existing knowledge and capabilities to develop new business innovation and, eventually, contribute to the strategic renewal of a firm’s competitive advantage (Corbett et al. 2013; Hill and Georgoulas 2016). That way, companies may possibly develop dynamic capabilities in their management practices and consequently entrepreneurial activities for continuous innovation and strategic renewal, enabling them to create and advance new combinations of resources through simultaneous exploration and exploitation (Eisenhardt and Martin 2000; Ireland and Webb 2007; Madsen 2010). Therefore, as CV can be associated with innovation and the strategic renewal of competitive advantage as a DC, but needs to be differentiated along distinctive features in the processes and routines of new business creation determining its organizational setup, as the following synthesis of CV through the lens of DC shows (Narayanan et al. 2009).

Examining the potential *functional domain* of CV as a DC, the analysis confirms the described multitude of different corporate venturing modes with the described new CV modes complementing the more traditional internal corporate venturing functions such as innovation, R&D and new business development (Dushnitsky and Birkinshaw 2014; Gutmann 2019; Hill and Georgoulas 2016; Kanbach and Stubner 2016; Narayanan et al. 2009). Hereby, the differentiation of these functional domains is not always clear, as terms are often used ambiguously, and different combinations are possible (Hill and Birkinshaw 2006). Therefore, the DC dimension of functional domains may include some corporate venturing modes in addition to traditional R&D and new product development, but their specific integration into the DC framework depends on the actual impact of the respective function on the company’s resource base (Alänge and Steiber 2018; Enkel and Sagmeister 2020).

An investigation of the *types of processes* in CV according to the DC framework confirms that CV can appear as a DC if it fulfils its entrepreneurial potential. This may involve sensing by recognising new problems, needs and opportunities; seizing by selecting and (re-) directing resources for new business models; and even transforming towards future business by reshaping the organizational structures and ordinary capabilities to adapt to new business opportunities (Augier and Teece 2009).

Turning towards the DC dimension of *hierarchies*, the potential for CV to fulfil all three DC types implies that it could act as a higher-level DC. However, this only applies if the CV function succeeds in influencing the parent firm’s lower-level ordinary capabilities, suggesting that it cannot be completely independent of the main organisation. This aligns with Winter (2003), who described DC levels as only locally defined. Thus, within a company with the ordinary capabilities of producing and selling goods or services, the creation of new business may be a higher-level capability. For an independent CV unit or lab, developing new products or services is an ordinary capability and, therefore, not a functional DC of the organisation.

This is why the dimension of *the focal unit of analysis* is especially critical for the integration of corporate venturing in the DC framework. The development and realisation of new business may take place across focal units at different levels of the organisation, such as in the corporate strategy that the top management team drives, business divisions, CV units, project teams, individual corporate ventures or individual members of the organisation (Zahra 1991). Indeed, the analysis confirms that scholars have examined corporate venturing in these very different focal units, all of which may be more or less aligned (Gibson and Birkinshaw 2004; Hill and Birkinshaw 2006, 2008).

**Fig. 5:**
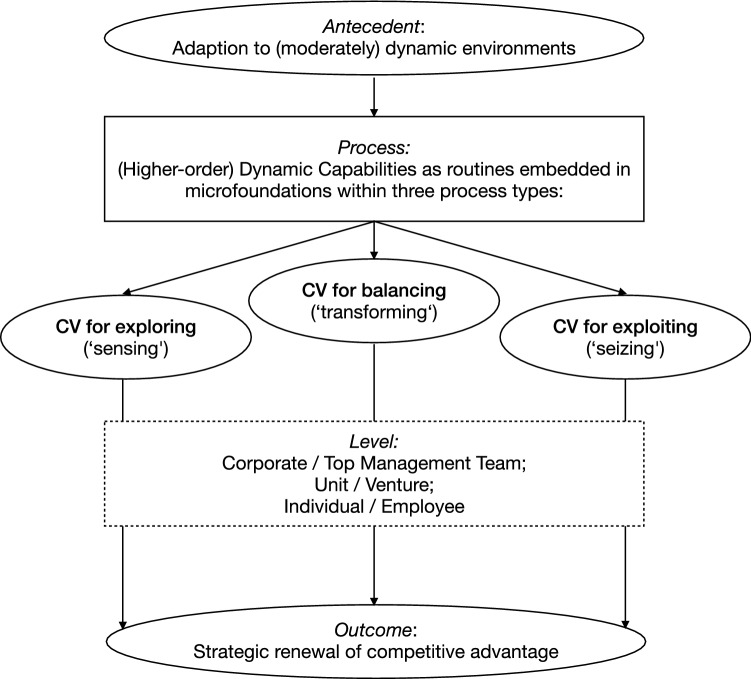
The DC view of CV for OA

Thus, to understand CV as a DC and examine its role in organizational ambidexterity, we must look at corporate venturing processes as either sensing or seizing on a lower order or transforming on a higher order. Moreover, the focal unit of analysis at hand and the corresponding CV mode that describes its specific function in the firm may influence those processes (see Fig. 5) (Hill and Georgoulas 2016). Suggesting such different possible roles for CV finally leads to answering the question of how CV may be linked to exploring, exploiting or balancing both, possibly making it a DC for the strategic renewal of firms competitive advantage.

### Corporate venturing for organizational ambidexterity as a dynamic capability

Many of the examined works differentiate between or focus on specific CV modes and focal units of analysis. This study remains agnostic on those dimensions, addressing instead the different sensing, seizing and transforming capabilities of various CV setups to explore, exploit or balance both. It probes these capabilities as a basis for assessing (without biased preselection) the possible roles and approaches of corporate venturing for organizational ambidexterity through the lens of DCs for an organizational and strategic point of view. From this perspective, the analysis confirms that corporate venturing can represent a means to capture the firm’s efforts to exploit current and explore new competitive advantages, which allows further integrating CV as a DC in the concept of OA (Burgers and Jansen 2008; Ireland and Webb 2007; Kuratko and Audretsch 2013).

**Fig. 6:**
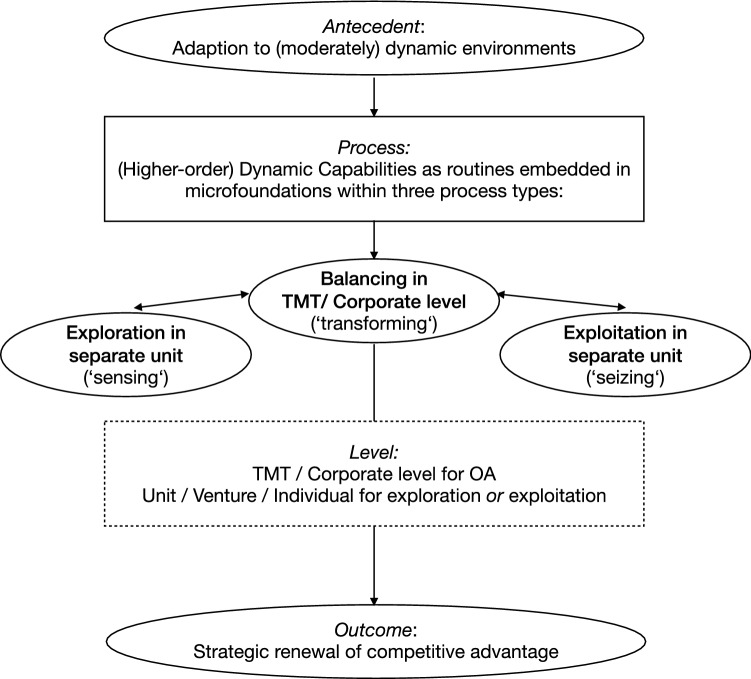
The DC view of CV for OA as trade-off

However, the exact role that CV can play for OA remains unclear across the literature. As stated in this study, the simultaneous balancing of exploration and exploitation can surface as a critical DC but may not be possible to achieve within one organizational function or unit, depending on the predominant logic. Following the ‘trade-off’ school of thought, CV and other organizational functions would focus on either sensing (exploring) or seizing (exploiting) as lower-order capabilities. The critical balance of both would be achieved as a higher-level dynamic capability on the corporate level within the top management team, or not at all (see Fig. 6). Indeed, many CV typologies and classifications that tend to differentiate between ‘exploring’ and ‘exploiting’ CV modes reflect this view, thereby neglecting the possibility of balancing both within a CV unit (e.g. Gutmann 2019; Hill and Birkinshaw 2008; Jansen et al. 2006). That way, new business innovation as exploring and operational business as exploiting oppose each other, with the clear separation of the CV function from the core business to overcome the OA tension, becoming almost axiomatic (Christensen 1998; Leten and Van Dyck 2012; Magnusson and Martini 2008). A surprising result in this predominant view is that CV would not act as a higher-level DC in this case, which may explain the often-missing strategic impact on the parent organisation of many CV modes in practice.

However, as the ‘paradox’ school of thought on OA suggested, the analysis of the CV literature also shows the potential for complementary exploration and exploitation in the CV function, thus making it a candidate to enable OA as a higher-level DC. Using this logic, CV units and other organizational entities engage not only in processes of sensing and seizing but especially transforming, enabling the paradox of simultaneous exploration and exploitation. This view appears most prominently in the literature that focuses on organizational links and interface mechanisms or (the context of) individual employee and middle-manager behaviour, rather than on structural separation and top management team routines (e.g. Farjoun 2010; Jansen et al. 2009). For instance, Burgers and Jansen (2008) differentiated between formal and informal integration, as well as organizational and top management-team integration mechanisms, clearly going further than the often-mentioned integration of exploration and exploitation by the top management team and an overarching vision (O’Reilly and Tushman 2011). Raisch (2008) described these phenomena as inter-organizational activities and mentioned CV explicitly as one example that can enable both explorative and exploitative knowledge processes.

**Fig. 7:**
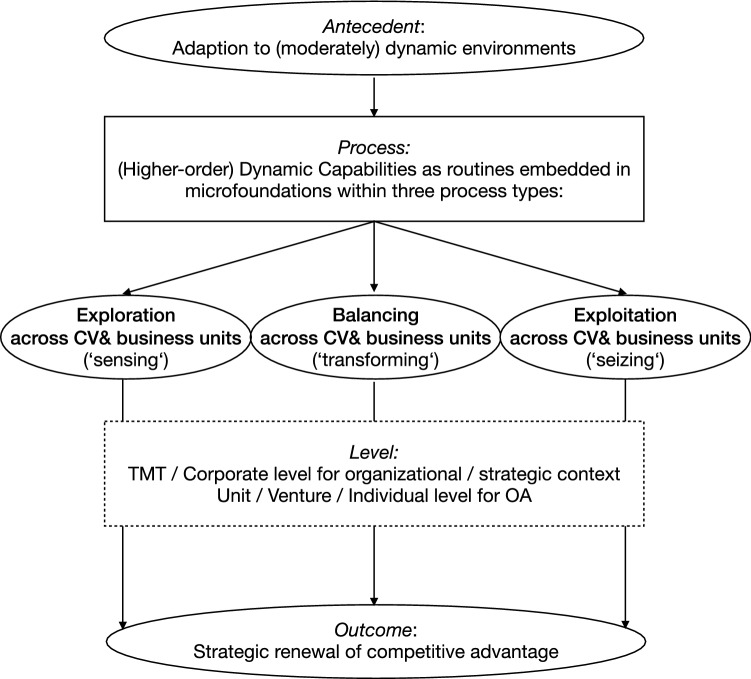
The DC view of CV for OA as paradox

While these examples already point towards tighter integration of exploration and exploitation on different levels, Hill and Birkinshaw (2014) in particular suggested more specifically that CV units could directly enable OA by covering both exploration and exploitation, while Rossi et al. (2019a) examined the same issue for corporate venture capital units, both confirming that OA may exist at different levels of the organisation and not only on the corporate level. While this is mostly based on specific balancing processes, interfaces and mechanisms, this view also aligns with the findings of Gibson and Birkinshaw (2004), describing the possibility of contextual OA, in which individuals in organizational units may be empowered to balance explorative and exploitative activities on their own. In any of these cases, organizational ambidexterity with the transforming dynamic capability of balancing exploration and exploitation would take place not only at the top management level of the organisation but also at lower levels between units, within units or from individual employees (see Fig. 7).

All in all, the analysis shows that, distinct from most common CV taxonomies and typologies, CV may enable OA as a DC with different roles and approaches, depending on its ability to provide not only sensing and seizing but also transforming capabilities that balance exploration and exploitation. This suggests a potentially more strategic role for corporate venturing processes and units, clearly confirming the relevancy of examining the link of CV with OA through the lens of DC (Rigtering and Behrens 2021). By unifying these concepts in the analysis, we can therefore suggest an integrated framework to demonstrate the possibilities of CV for OA, based on relevant dimensions of the DC concept, as a fundament for further research and reflection on the practice of strategic management.

## Results: toward an integrated framework of CV for OA as a DC

The analysis underlines not only the relevance of building an integrated view of corporate venturing and organizational ambidexterity, as stated in the research question, but it also leads to possible concrete organizational setups by clearly defining and differentiating CV and OA along selected dimensions of the DC framework. Most importantly, the analysis indicates here that CV can enable OA by simultaneously balancing exploration and exploitation and thus act as a DC to renew a firm’s competitive advantage in the form of strategic corporate venturing (see Appendix V). Enabling ambidexterity thus critically influences the role of CV as a potentially higher-order dynamic capability, in contrast to a lower-order capability in which CV does not enable ambidexterity directly, i.e. requiring a top management team or other means of structural organizational ambidexterity to balance exploration and exploitation. To provide these findings in a concise way and fulfil the research objective to conceptualise possible organizational setups of CV for OA, the following integrated framework and subsequent aggregation and interpretation of relevant empirical studies build on the dimensions of OA and DC as identified and described in the systematic review and analysis (see Appendix V,VI):

From the ambidexterity perspective, the ‘trade-off’ versus ‘paradox’ logic differentiates corporate venturing activities that either explore *or* exploit (trade-off) from both exploring *and* exploiting (paradox) modes. We hereby build on the main results of the analysis, showing that a trade-off logic usually requires a balancing of exploration and exploitation on the system or organizational level as individuals or single units cannot manage both simultaneously. This would suggest a rather operative, lower-order CV focusing on sensing or seizing activities, probably not acting as a dynamic capability, as it requires moderation by higher-order (dynamic) capabilities. In contrast, the paradox logic describes the possibility of complementary exploration and exploitation within individuals or units of the organisation, especially enabled by supporting structures, processes, interfaces, cultures or other means. This would lead to a more strategic role for CV as a higher-order (dynamic) capability, possibly incorporating relevant sensing, seizing and transforming processes.

In both dimensions of the ambidexterity logic, the dynamic capabilities logic provides possibilities for further differentiation. From the examined DC dimensions, the functional domain is already considered in the overall framework with the focus on CV, and the hierarchies of capabilities as well as the types of processes are integrated into the OA logic as described above. Therefore, particularly the focal unit of analysis offers more distinction, describing that DCs might be embedded in individual/team behaviours or organizational processes and routines, which can be seen in both CV and OA. Here, CV can be moderated by a specialised unit in a focused manner or be dispersed across individuals in the organisation, thus being present in organizational structures and processes or individual routines and behaviours. Likewise, OA can be balanced by behaviours and routines of individual employees or top managers, or it may emerge in specific organizational structures and processes that separate or link distinct units.

**Fig. 8:**
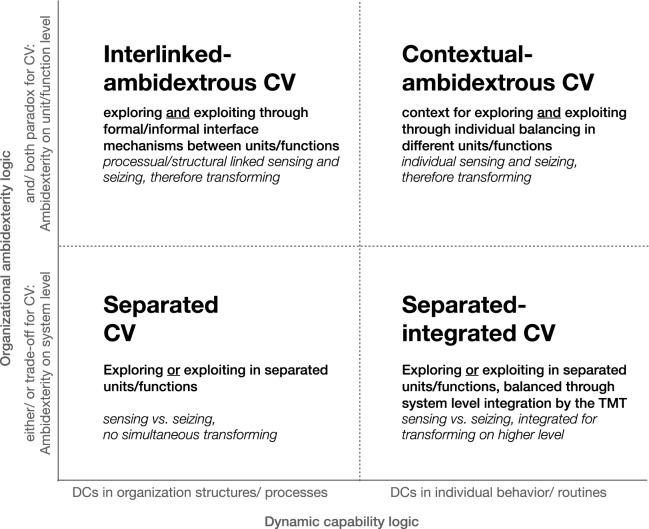
Integrated framework of CV for OA as a DC

The combination of these OA and DC logics for CV consequentially leads to an integrated framework with four possible approaches of CV for OA, each further detailed with additional DC and OA considerations (see Fig. 8). With the subsequent aggregation and interpretation of relevant empirical CV and OA studies in these dimensions, the resulting integrated framework fulfils the research objective to transcend the results of individual studies by suggesting possible new approaches for CV and OA in established firms for further examination (see Appendix VI).

### Separated corporate venturing

In the ‘trade-off’ dimension, *separated CV* describes the strict separation of explorative and exploitative activities in the organizational structure, e.g., through a spin-off of a corporate venture, separate alliances or other general forms of outsourcing (Baden-Fuller and Volberda 1997; Chesbrough 2000; Lavie and Rosenkopf 2006; Raisch and Birkinshaw 2008). Here, balancing exploration and exploitation activities may be achieved over time at a system level, but not simultaneously, as no clear links exist at an individual or organizational level. In regard to the analysis, separated CV can be seen as a lower-order capability as sensing and seizing are conducted in different units or functions, while a transforming capability is not specifically defined. This suggests that in this setup, not only does CV not act as a dynamic capability, but the entire organisation may not necessarily possess the dynamic capabilities necessary to simultaneously explore and exploit to address the ambidexterity challenge.

Consequently, examining the ambidexterity literature produced little empirical support for this approach, and the few examples largely stem from a time when OA theory was still in its early development phase (see Appendix VI). This particularly includes the study of three CV units in the disc-drive industry by Christensen and Bower (1996), who strongly influenced the debate early on by presenting successful cases of independent corporate venturing. Although the examinations that followed often confirmed the successful exploration in separate CV units, simultaneous exploitation was rarely achieved, demonstrating that the missing alignment and, therefore, the missing ambidexterity eventually hindered sustainable success. Until the 1990s, examples of these findings included various established firms, such as Xerox PARC (Heracleous et al. 2017); IBM, Eastman Kodak and Philips (Baden-Fuller and Volberda 1997); C&W and Telia (Covin and Miles 2007); USA Today (Tushman et al. 2010); and Unilever (Jones and Kraft 2004) (see Appendix VI). Even today, such cases still appear—e.g., new forms of corporate accelerators that fail to connect external start-ups with the core business (Moschner and Herstatt 2017).

### Separated-integrated corporate venturing

As an elaboration of the original separated ‘trade-off’ approach, separated-integrated corporate venturing addresses the missing ambidexterity by providing a link back to the core business (Benner and Tushman 2003). Thus, from an OA logic, explorative and exploitative activities are still strictly separated in the organizational structure following the trade-off logic, describing the difficulty to enable both simultaneously within the main organisation at an individual or unit/function level. However, this emerging organizational ambidexterity concept offers the workaround of structural ambidexterity with a larger body of empirical evidence examining individual routines and behaviour and an overarching corporate vision at the top management level as means to balance the organizationally separated activities (Tushman et al. 2010). Consequently, different functions such as CV still only cover sensing or seizing processes, while transformation or reconfiguration happens mostly through integration efforts in corporate management. Therefore, CV again acts as a lower-order capability, while the dynamic capability of reconfiguring assets and balancing exploration and exploitation takes place within the bigger system at the top management level at best. Integrated-separated CV hence aims to realise the best of both worlds by letting separate CV units or functions explore new opportunities, independent of restrictions from the current core business, while the corporate level ensures that these activities stay connected to overall strategies for later exploitation (Farjoun 2010; Taylor and Helfat 2009). As this approach expresses the origins of structural ambidexterity in the ‘trade-off’ school, the literature offers various examples from empirical examinations (see Appendix VI).

Notably, Tushman and O’Reilly (1996) advanced this approach with several studies investigating it in such companies as HP, J&J and ABB, a later stage analysis of the USA Today case (O’Reilly and Tushman 2004), or at IBM, Cisco and Middleware (O’Reilly et al. 2009; O’Reilly and Tushman 2011). Even as alternative approaches for OA emerge, separated-integrated CV remains a relevant concept in the current literature—for instance, in the examination of Nestlé Nutrition (Birkinshaw et al. 2016) and at multiple corporate accelerators (Kanbach and Stubner 2016), incubators (Kupp et al. 2017) or spin-alongs (Mahdjour and Fischer 2014). In a recent multiple case study of external corporate venturing, Enkel and Sagmeister (2020) recognised that only the combination of multiple CV modes could act as a dynamic capability. Likewise, many of the examples depict successes that heavily depend on top management’s ability to provide the DC of transforming to balance exploration and exploitation, limiting its practical applicability for successful OA and CV in other firms, as individual behaviours and routines of top management teams are difficult to identify and build or copy. Thus, it remains challenging to achieve ambidexterity within the trade-off approach as a universal dynamic capability.

### Contextual-ambidextrous corporate venturing

To address the challenges of the trade-off approach, alternative logics of corporate venturing have attempted to enable ambidexterity differently following the ‘paradox’ school of thought. The introduction of *contextual-ambidextrous CV*, especially by Gibson and Birkinshaw (2004), questioned the need for structural separation. In contrast, it is recognised here that individual employees may be able to balance exploration and exploitation simultaneously if required behaviour and routines are enabled by suitable contextual factors such as the organizational culture and strategy. Consequently, ambidexterity could be achieved here at unit or project levels within the organisation instead of a higher system level. Following this logic, employees and (middle) managers in the corporate venturing programme engage in both sensing and seizing processes simultaneously, and thus potentially also in transformation and renewal. Hence, CV activities could present a higher-order dynamic capability through individual behaviour and routines in the main organisation.

Indeed, contextual CV is now recognised as an alternative approach towards OA, which can also be found in the identified empirical examinations (see Appendix VI). However, the body of literature is limited in our investigation, probably because such contextual CV often focuses on rather unstructured CV programmes or the organisation as a whole, thus not being easy to identify and examine from the outside. In the identified studies, employees are usually classified as intrapreneurs, either with formal or informal support, to explore and exploit new ideas, e.g. at 3 M and P&G (Miles and Covin 2002), Skandia AB and Ericsson (Covin and Miles 2007), Google (Wolcott and Lippitz 2007), a selection of leading design firms (Andriopoulos and Lewis 2009), or at Tencent and Alibaba (Du and Chen 2018). For instance, a recent single case study of Samsung Creative-Lab and a subsequent multiple case study of the program’s ventures illustrate contextual-ambidextrous CV with exploration and exploitation conducted by employees, resulting in independent spin-offs that diversified the overall business portfolio of Samsung and thus potentially contributed to its strategic renewal and competitive advantage through a structured CV setup (Shin and Cho 2020).

Even though all these examples show the practicality of contextual-ambidextrous CV, the evolving opportunities can often not be planned, thus limiting its contribution to the organizationally relevant strategic renewal of the competitive advantage, which is usually a precondition for the recognition of a dynamic capability. Likewise, individual behaviour and routines, which are enabled especially by contextual factors such as company culture and require a specific skill set of employees, may be difficult to replicate as a reproducible way to address the ambidexterity challenge. Thus, contextual-ambidextrous CV therefore may or may not act in the sense of strategic corporate venturing, enabling OA as a DC.

### Interlinked-ambidextrous corporate venturing

In contrast, *interlinked-ambidextrous CV* offers a more structured ‘paradox’ approach for corporate venturing to enable organizational ambidexterity, which a larger and still growing body of literature demonstrates (see Appendix VI). Here, a CV unit or function deliberately engages in sensing and seizing process to explore and exploit relevant opportunities for the organisation, thus potentially resulting in transforming it for the strategic renewal of the competitive advantage as a dynamic capability. In contrast to the previous approach, this higher-order dynamic capability of balancing exploration and exploitation is here not predominantly represented in individual behaviours and routines but rather embedded in organizational structures and processes within and between units and functions, resulting in ambidexterity to be achieved at this level rather than in the corporate management or system alone (Hill and Birkinshaw 2014; Jansen et al. 2009). Consequently, the approach of interlinked-ambidextrous CV seems to offer the greatest potential for CV to enable OA as a DC.

Interestingly, while this approach is reflected in various contemporary forms of CV, it also follows the early pivotal works of Burgelman (1983, 1984a, 1984b) on CV, whose framework implied that opportunities with strategic importance and operational relatedness (thus, relevant resources to leverage) should not be completely separate but should maintain relevant links. Later on, Hill and Birkinshaw (2008, 2014) confirmed this suggestion in their examination of mechanisms for OA at the business-unit level, resulting in CV units that can develop an ambidextrous orientation by building linkages with internal and external actors. Further, a surprisingly big and growing body of literature with various empirical studies also confirms the possible integration of OA in the management of specific organizational units (see Appendix VI). This includes, for instance, Burgelman and Välikangas (2005), Tidd and Taurins (1999) and Chesbrough (2000, 2002), with different case studies on Microsoft, Lucent, Cisco, Intel and Panasonic. Complementary cases, in which the required organizational linkages are often specifically examined, include the single case studies from Martin and Eisenhardt (2004), Vanhaverbeke and Peeters (2005) and McGrath et al. (2006).

More broadly, these findings could be extended in a more general sense through various multiple case studies, showing, for instance, ‘hybrid designs’ (Westerman et al. 2006), ‘reciprocal causality’ and ‘CV as a strategy’ (Covin and Miles 2007), or specific types of linking mechanisms (O’Connor and DeMartino 2006; Raisch and Birkinshaw 2008; Taylor and Helfat 2009). Based on this groundwork, similar approaches are apparent within newer specific CV modes, such as the spin-along ventures that Michl et al. (2012) describe, the ‘outside-in’ model of Siemens TBB and the ‘inside-out’ model of SAP Hana and PayPal (Weiblen and Chesbrough 2015), the ‘venture-client’ model at BMW (Gimmy et al. 2017) and WAYRA (Gutmann et al. 2020), ‘corporate incubators’ (Gassmann and Becker 2006) and ‘open innovation’ vehicles such as GE FirstBuild, the Electrolux Open Innovation and Lantmännen Greenhouse Accelerator (Alänge and Steiber 2018) and the emerging ‘digital innovation labs’ (Holotiuk and Beimborn 2018). Leten and Van Dyck (2012) also confirmed these interlinked mechanisms in the cases of GlaxoSmithKline, SNCF and Alcatel-Lucent, while other scholars have identified them in processes over time, such as in the front-end of innovation (Cantarello et al. 2012) or along the ‘graduation process’ of CVs (Raisch and Tushman 2016). In yet another recent example, Gutmann et al. (2019) illustrated a potentially interlinked-ambidextrous setup in the single case study of the SAP industry 4.0 start-up programme, in which external start-ups were specifically chosen based on their fit with the core offer and the deliberate exploitation of opportunities with direct integration support into the SAP portfolio. This underlines that especially new contemporary forms may play a major role in the pursuit of interlinked-ambidextrous CV.

Indeed, by focusing on various organizational interfaces and processual linking mechanisms, these studies clearly question the ‘trade-off’ approach, suggesting that CV units with closer relationships to the core business may have more sustainable success (O’Hare et al. 2008). However, more often than not, this finding of an ambidextrous approach is often rather a by-product of the CV examination, which only comes to light in the synthesis of various empirical papers such as the present systematic review. This suggests that even though interlinked-ambidextrous CV appears in many different CV modes, a holistic empirical view of how CV may enable OA as a DC is still missing, and the subject could profit from a more deliberate examination of the phenomenon in subsequent empirical studies (Rossi et al. 2019a).

As a final result, the proposed integrated framework clearly presents the possibility that corporate venturing can directly enable organizational ambidexterity as a dynamic capability, and therefore contribute to the strategic renewal of established firms’ competitive advantage either by contextually supporting ambidextrous individual behaviours or by providing interlinked structures and processes. Specifically, the latter can be seen as strategic corporate venturing, in which the creation of new business also acts as a means for strategic entrepreneurship within the domain of corporate entrepreneurship by engaging in simultaneous opportunity- and advantage-seeking behaviours, resulting in the relevant change of the main organisation to sustain future profitable growth.

## Discussion and conclusion

Through the conceptual integration in the resulting novel framework, this systematic literature review presents a useful foundation to deliberately consider specific strategic roles and organizational setups of CV for OA as a DC, promoting further research to enhance the concepts of organizational ambidexterity and dynamic capabilities and providing new insights into possible corporate venturing modes and characteristics.

First and foremost, the analysis and results provide an interconnected view of CV, OA and DC, therefore elaborating on all three theories by focusing on their interfaces. As presented, current academic debates already have discussed specifically each singular interface in regard to OA and DC (i.e. Birkinshaw et al. 2016; Snehvrat et al. 2018), CV and DC (i.e. Enkel and Sagmeister 2020; Hill and Georgoulas 2016) and CV and OA (i.e. Gutmann 2019; Leten and Van Dyck 2012). However, while the parallel emergence of these concepts in theory and practice and clearly existing interconnections call for a better understanding of this interrelatedness, an integrated view was still missing. We believe that our contribution can serve as a foundation to further elaborate the intersections of all three concepts in the future, specifically in terms of empirical research. In that sense, the resulting integrated framework may serve as a categorisation scheme by harmonising, simplifying and organising the interconnected phenomena of CV, OA and DC to highlight the potential interrelations between them and their overall objective of creating sustainable competitive advantage for established firms in a complex, fast-changing environment (Christensen and Carlile 2005).

As a basis for future research, the study not only could concretise the interdependency of the different theoretical methods. It also presents various setups of corporate venturing for organizational ambidexterity that indicate the possibility of a more strategic role for CV as a dynamic capability, in contrast to existing typologies and taxonomies that imply a purely explorative (or exploitative) role for CV (e.g. Gutmann 2019; Hill and Birkinshaw 2008; Kanbach and Stubner 2016). This specifically extends current debates on the distinction of corporate venturing and strategic entrepreneurship activities within the domain of corporate entrepreneurship, showing that ambidextrous CV does not only result in new business for the firm but may also directly engage in strategic or organizational change (Hill and Georgoulas 2016; Sakhdari 2016). This can, therefore, possibly lead to strategic corporate venturing in which the creation of new business also supports the strategic renewal of a firm’s competitive advantage through simultaneous opportunity-seeking (exploration) and advantage-seeking (exploitation) behaviour.

Examining more specifically such interrelated forms of corporate venturing and strategic entrepreneurship in the future could add significant insights to the concept of corporate entrepreneurship. This is especially relevant in practice, as firms are often struggling to find the right employment of these concepts, particularly because distinctions are not as clear in practice as they seem in theory. Based on the examined publications, an interesting avenue should be the examination of contemporary corporate venturing and innovation endeavours to shed light on their non-financial, strategic benefits (Dushnitsky and Birkinshaw 2014; Hill and Georgoulas 2016; Shankar and Shepherd 2019). This may also be applicable in the research on specific organizational change phenomena such as digital transformation, where entrepreneurial (digital) units and their (middle) managers may play a decisive role (Nadkarni and Prügl 2021).

In addition to providing a more clearly differentiated picture on this matter, the presented synthesis and classification of selected empirical studies in the possible CV setups also show the relevance of different (dynamic) capabilities for sensing, seizing and potentially transforming to explore, exploit and balance them in various CV modes. Here, the examined literature provides convincing evidence that CV can and should be differentiated by its (dynamic) capabilities to explore and/or exploit, but a deeper, empirically derived classification of CV types based on these dimensions is still lacking. Therefore, these novel findings may be a foundation for further research into contemporary CV modes, with respect to their abilities to sense, seize and transform, to achieve organizational ambidexterity and, thus, create a new sustainable competitive advantage as a dynamic capability. Here, future (empirical) studies may examine the potentially more strategic role of corporate venturing and the innovation function for firms that must adapt to changing business environments, besides empirically extending and confirming the proposed integrated framework. Following this proposed research direction would not only stipulate the possibility of harmonising currently variable CV modes with common strategic roles but also may provide new insights into the concrete applications of OA as a DC in the structures and processes of established companies.

This examination furthermore also reflects on the dynamic capabilities framework itself. Here, our systematic review specifies the proposed DC dimensions with clearly defined use cases of CV for OA. In that sense, the systematic review confirms the relevancy of the DC framework in strategic management again and points out the required rigour applicable to avoiding oversimplified classifications of various activities as dynamic capabilities. Accordingly, more specific applications may further enhance the DC theory in the future, especially regarding higher- and lower-order dynamic capabilities for OA in CV and other organizational functions. In this context, the presented integration of different DC dimensions for a coherent structure throughout the analysis should be a useful contribution following the call of Schilke et al. (2018) for the further integration of different DC dimensions, which should also help to apply the context more holistically in practice.

Further, the present examination has not only relevant implications in the domain of corporate entrepreneurship and dynamic capabilities but also provides new insights into the current debate around ambidexterity in the domain of organizational science, presenting more clearly where and how the challenge of simultaneously balancing exploration and exploitation can be solved, which is often vaguely formulated (O’Reilly and Tushman 2013). While the current debate already considers different ways to address the ambidexterity challenge, such as structural, sequential or contextual ambidexterity, the results suggest a more granular differentiation based on various DC attributes to describe and examine how OA might manifest itself, for instance, in different focal units of analysis, functions or types of processes (Simsek et al. 2009). Furthermore, the results clearly support the emerging paradoxical view through which OA can be achieved within individual behaviours and routines or cross-organizational structures and processes through the identification of empirical studies for interlinked-ambidextrous and contextually-ambidextrous CV, challenging the clear distinction between exploration and exploitation in the trade-off approach (Papachroni et al. 2014). We would therefore strongly recommend considering the possibility of simultaneous exploration and exploitation in future examinations of CV activities, as well as in comparable innovation activities such as research & development (Boiko 2021).

Concerning preferred methodologies, we would argue to further elaborate on the intersections of CV, OA and DC with qualitative studies. Although some case studies could be identified for these phenomena, most did not deliberately position themselves at these intersections, making any insights towards CV for OA as a DC rather a by-product as opposed to a reproducible finding. Qualitative studies focusing on these aspects could, therefore, further develop the foundation for more quantitative research, which may then confirm some of the results (Christensen and Carlile 2005).

All in all, these relevant theoretical contributions confirm the view that systematic literature reviews not only provide a broad overview of the topic but may even result in novel theoretical constructs that lead to new or enhance existing directions in the research area (Kraus et al. 2020). In addition to these theoretical contributions, the systematic literature review can also provide more clarity for the practice of CV and strategic management. Indeed, today, almost every organisation finds itself in at least moderately dynamic, but often even radically changing, business environments. A clearer view of possible organizational approaches to CV that simultaneously explore and exploit can be of use in building the required DCs. Here, the present research especially provides a sound perspective on the possible strategic role for CV for OA as a DC, instead of limiting it solely to an explorative or exploitative ‘side function’ in the organisation.

With CV activities again reaching new heights in practice, with new, often digital innovation hubs, labs, incubators and accelerators, their role is an important topic for responsible managers to consider, too often remaining unclear today (Holotiuk and Beimborn 2018). Consequently, failure rates of these organizational vehicles are still high, while established companies struggle with corporate entrepreneurship and strategic renewal, despite several waves of CV practice and research. Even though established firms continue to experiment with new- and old-format CV modes, and the field shows some maturation, the overall success rate of such activities continues to be highly variable, as the arrangements are often short-lived and yield limited results (Dushnitsky and Birkinshaw 2014; Jones and Kraft 2004). A critical view reveals that most CV activities still fail to generate impact by reaching strategic innovation goals. Consequently, many high-profile companies, including Volkswagen, Yahoo, Turner/Warner Bros and Coca-Cola, have closed down many CV activities, despite the overall trend (Gimmy et al. 2017). Thus, establishing new businesses in a corporate structure seems to remain one of the most difficult challenges in the strategic management of firms, mirroring the complex tension of simultaneously exploring and exploiting (Raisch and Tushman 2016).

In this context, the differentiated view on CV for OA through the lens of DC can help managers to not only reflect the CV setups currently in use but also put them into the larger strategic management context to assess if and how they support OA to build future sustainable competitive advantage. Here, the potentially more strategic role of CV positions corporate innovation and venturing units at the heart of organizational strategy, instead of as a separate side function, and might create the basis for a new organizational design that overcomes separation and centralisation to integrate strategic innovation and business units further. That way, the findings not only contribute to the theories of OA and DC but, more specifically, suggest new concepts for CV that are also relevant for management practice. Likewise, managers who are concerned about broader organizational and strategic development may find new insights into the application of the DC framework in practice and new perspectives on how to simultaneously pursue exploration and exploitation at different levels of the organisation. Here, using more elaborate definitions of the different dynamic capabilities’ dimensions may be especially useful for practitioners in the future. For example, the elaborated but not yet clearly empirically examined model of sensing, organising, value capturing, and renewing may be of help to further operationalise such dimensions in practice (Teece et al. 2020).

In that way, the systematic literature review fulfils its objective of not only contributing to the academic debate but also supporting better decision-making for policymakers and managers (Kraus et al. 2020). However, both the theoretical and practical contributions are subject to the limitations of the chosen methodology and the constraints of the research project (Tranfield et al. 2003). Although systematic literature reviews are largely seen as strong evidence, personal bias cannot be eliminated completely, even with a transparent sampling and analysis process. Despite discussing the methodology and preliminary results with fellow researchers at relevant conferences, workshops and seminars, resources—especially time—still prevented us from reaching conclusive evidence (Kraus et al. 2020). In the conceptualisation of an integrated framework, confusion and contradiction are typically the norm, as other scholars might find different logics and coding schemes useful (Christensen and Carlile 2005). However, we still find the contributions to be relevant, especially considering that the elimination of bias is not expected to be possible within the domains of entrepreneurship and strategic management as social sciences (Tranfield et al. 2003). However, the resulting integrated framework should not be seen as a new truth but rather as a substantial contribution to the existing literature that describes the specifically targeted attributes of the examined phenomena. In that sense, the proposed model can explain some anomalies of existing frameworks—for instance, in bridging the ‘trade-off’ and ‘paradox’ views—but may also suffer from anomalies of its own that subsequent studies should re-examine (Christensen and Carlile 2005).

With these limitations in mind, the research objective of examining the connection of CV with OA and identifying different setups accordingly could be fulfilled, but we cannot provide a final answer on the topic. Instead, the hope is that the presented integrated framework will serve as an inspiring starting point in the desired direction, offering researchers and practitioners new perspectives for examining and realising dynamic capabilities for the strategic renewal of the firm with strategic corporate venturing for organizational ambidexterity.

## Supplementary Information

Below is the link to the electronic supplementary material.

Supplementary file1 (PDF 62 KB)

Supplementary file2 (PDF 81 KB)

Supplementary file3 (PDF 74 KB)

Supplementary file4 (PDF 82 KB)

Supplementary file5 (PDF 83 KB)

Supplementary file6 (PDF 150 KB)

## References

[CR1] Adner R, Helfat CE (2003). Corporate effects and dynamic managerial capabilities. Strateg Manag J.

[CR2] Agostini L, Nosella A, Filippini R (2016). Ambidextrous organisation and knowledge exploration and exploitation: the mediating role of internal networking. Int J Bus Innov Res.

[CR3] Ahuja G, Lampert CM, Tandon V (2008). Moving beyond Schumpeter: management research on the determinants of technological innovation. Acad Manag Ann.

[CR4] Alänge S, Steiber A (2018). Three operational models for ambidexterity in large corporations. Triple Helix.

[CR5] Almahendra R, Ambos B (2015). Exploration and exploitation: a 20-year review of evolution and reconceptualisation. Int J Innov Manag.

[CR6] Ambrosini V, Bowman C (2009). What are dynamic capabilities and are they a useful construct in strategic management?. Int J Manag Rev.

[CR7] Anand J, Oriani R, Vassolo RS (2010). Alliance activity as a dynamic capability in the face of a discontinuous technological change. Org Sci.

[CR8] Andriopoulos C, Lewis M (2009). Exploitation-exploration tensions and organizational ambidexterity: managing paradoxes of innovation. Organ Sci.

[CR9] Augier M, Teece DJ (2009). Dynamic capabilities and the role of managers in business strategy and economic performance. Organ Sci.

[CR10] Aguinis H, Ramani RS, Alabduljader N (2018). What you see is what you get? Enhancing methodological transparency in management research. Acad Manag Ann.

[CR11] Baden-Fuller C, Volberda HW (1997). Strategic renewal: how large complex organizations prepare for the future. Int Stud Manag Organ.

[CR12] Barney J (1991). Firm resources and sustained competitive advantage. J Manag.

[CR13] Barreto I (2010). Dynamic capabilities: a review of past research and an agenda for the future. J Manag.

[CR14] Battistini B, Hacklin F, Baschera P (2013). The state of corporate venturing: insights from a global study. Res Technol Manag.

[CR15] Benner MJ, Tushman ML (2003). Exploitation, exploration, and process management: the productivity dilemma revisited. Acad Manag Rev.

[CR16] Bierwerth M, Schwens C, Isidor R, Kabst R (2015). Corporate entrepreneurship and performance: a meta-analysis. Small Bus Econ.

[CR17] Birkinshaw J, Batenburg R, Murray G (2002). Venturing to succeed. Bus Strategy Rev.

[CR18] Birkinshaw J, Hill S (2005). Corporate venturing units: vehicles for strategic success in the new Europe. Organ Dyn.

[CR19] Birkinshaw J, Zimmermann A, Raisch S (2016). How do firms adapt to discontinuous change? Bridging the dynamic capabilities and ambidexterity perspectives. Calif Manag Rev.

[CR20] Blindenbach-Driessen F, van den Ende J (2014). The locus of innovation: the effect of a separate innovation unit on exploration, exploitation, and ambidexterity in manufacturing and service firms. J Prod Innov Manag.

[CR21] Boiko K (2021). R&D activity and firm performance: mapping the field. Manag Rev Q (online).

[CR23] Breslin D, Gatrell C, Bailey K (2020). Developing insights through reviews: reflecting on the 20th anniversary of the International Journal of Management reviews. Int J Manag Rev.

[CR24] Burgelman R (1983). A model of the interaction of strategic behavior, corporate context, and the concept of strategy. Acad Manag Rev.

[CR25] Burgelman R (1984). Designs for corporate entrepreneurship in established firms. Calif Manag Rev.

[CR26] Burgelman R (1984). Managing the internal corporate venturing process. Sloan Manag Rev.

[CR27] Burgelman R, Välikangas L (2005) Internal corporate venturing cycles: a nagging strategic leadership challenge. Stanford GSB research paper, no. 1908

[CR28] Burgers H, Jansen J (2008) Organizational ambidexterity and corporate entrepreneurship: the differential effects on venturing, innovation and renewal processes. In: Zacharakis A (ed) Frontiers of entrepreneurship research 2008: proceedings of the 28th annual entrepreneurship research conference. Babson College, Chapel Hill, NC, pp 1–15

[CR31] Cantarello S, Martini A, Nosella A (2012). A multi-level model for organizational ambidexterity in the search phase of the innovation process. Creat Innov Manag.

[CR32] Chen Y (2017). Dynamic ambidexterity: how innovators manage exploration and exploitation. Bus Horiz.

[CR33] Chesbrough H (2000). Designing corporate ventures in the shadow of private venture capital. Calif Manag Rev.

[CR34] Chesbrough HW (2002). Making sense of corporate venture capital. Harv Bus Rev.

[CR35] Christensen CM (1997). The innovator’s dilemma.

[CR36] Christensen CM (1998). Why great companies lose their way. Across Board.

[CR37] Christensen CM, Bower JL (1996). Customer power, strategic investment, and the failure of leading firms. Strateg Manag J.

[CR38] Christensen CM, Carlile P (2005) The cycles of theory building in management research. Working paper series

[CR39] Collis DJ (1994). Research note: how valuable are organizational capabilities?. Strateg Manag J.

[CR40] Corbett A, Covin JG, O’Connor GC, Tucci CL (2013). Corporate entrepreneurship: state-of-the-art research and a future research agenda. J Prod Innov Manag.

[CR41] Covin JG, Miles MP (1999). Corporate entrepreneurship and the pursuit of competitive advantage. Entrep Theory Pract.

[CR42] Covin JG, Miles MP (2007). Strategic use of corporate venturing. Entrep Theory Pract.

[CR43] Du J, Chen Z (2018). Applying organizational ambidexterity in strategic management under a ‘VUCA’ environment: evidence from high tech companies in China. Int J Innov Stud.

[CR44] Dushnitsky G, Birkinshaw J (2014). Corporate venturing virtual special issue. Strateg Manag J.

[CR45] Easterby-Smith M, Lyles MA, Peteraf MA (2009). Dynamic capabilities: current debates and future directions. Br J Manag.

[CR46] Eisenhardt KM, Furr NR, Bingham CB (2010). Microfoundations of performance: balancing efficiency and flexibility in dynamic environments. Organ Sci.

[CR47] Eisenhardt KM, Martin JA (2000). Dynamic capabilities: what are they?. Strateg Manag J.

[CR48] Enkel E, Sagmeister V (2020). External corporate venturing modes as new way to develop dynamic capabilities. Technovation.

[CR49] Farjoun M (2010). Beyond dualism: stability and change as a duality. Acad Manag Rev.

[CR50] Fisch C, Block J (2018). Six tips for your (systematic) literature review in business and management research. Manag Rev Q.

[CR51] Frank H, Hatak I, Fayolle A, Wright M (2014). Doing a research literature review. How to get published in the best entrepreneurship journals.

[CR52] Gassmann O, Becker B (2006). Towards a resource-based view on corporate incubators. Int J Innov Manag.

[CR53] Gibson CB, Birkinshaw J (2004). The antecedents, consequences, and mediating role of organizational ambidexterity. Acad Manag J.

[CR54] Gimmy G, Kanbach D, Stubner S, Konig A, Enders A (2017) What BMW’s corporate VC offers that regular investors can’t. Harv Bus Rev. https://hbr.org/2017/07/what-bmws-corporate-vc-offers-that-regular-investors-cant. Accessed 23 Dec 2020

[CR56] Gupta AK, Smith KG, Shalley CE (2006). The interplay between exploration and exploitation. Acad Manag J.

[CR57] Gusenbauer M, Haddaway NR (2020). Which academic search systems are suitable for systematic reviews or meta-analyses? Evaluating retrieval qualities of Google Scholar, PubMed, and 26 other resources. Res Synth Methods.

[CR58] Gutmann T, Kanbach D, Seltman S (2019). Exploring the benefits of corporate accelerators: investigating the SAP Industry 4.0 Startup program. Probl Perspect Manag.

[CR59] Gutmann T, Maas C, Kanbach D, Stubner S (2020). Startups in a corporate accelerator: what is satisfying, what is relevant and what can corporates improve?. Int J Entrep Innov Manag.

[CR60] Gutmann T (2019). Harmonizing corporate venturing modes: an integrative review and research agenda. Manag Rev Q.

[CR61] Harzing AW, Alakangas S (2016). Google Scholar, Scopus and the Web of Science: a longitudinal and cross-disciplinary comparison. Scientometrics.

[CR62] Helfat CE, Finkelstein S, Mitchell W, Peteraf M, Singh H, Teece D, Winter SG (2007) Dynamic capabilities and organizational processes. In: Dynamic capabilities: understanding strategic change in organizations, 1st edn. Wiley-Blackwell, Malden, MA, pp 30–45

[CR63] Helfat CE, Peteraf MA (2009). Understanding dynamic capabilities: progress along a developmental path. Strateg Organ.

[CR64] Helfat CE, Winter SG (2011). Untangling dynamic and operational capabilities: strategy for the (N)ever-changing world. Strateg Manag J.

[CR65] Heracleous L, Papachroni A, Andriopoulos C, Gotsi M (2017). Structural ambidexterity and competency traps: insights from Xerox PARC. Technol Forecast Soc Change.

[CR66] Hill SA, Birkinshaw J (2006). Ambidexterity in corporate venturing: simultaneously using existing and building new capabilities. Acad Manag Proc.

[CR67] Hill SA, Birkinshaw J (2008). Strategy–organization configurations in corporate venture units: impact on performance and survival. J Bus Ventur.

[CR68] Hill SA, Birkinshaw J (2014). Ambidexterity and survival in corporate venture units. J Manag.

[CR69] Hill SA, Georgoulas S, Zahra S, Neubaum D, Hayton C (2016). Internal corporate venturing: a review of (almost) five decades of literature. Handbook of research on corporate entrepreneurship.

[CR70] Hitt MA, Ireland DR, Sirmon DG, Trahms CA (2011). Strategic entrepreneurship: creating value for individuals, organizations, and society. Acad Manag Perspect.

[CR71] Holotiuk F, Beimborn D (2018). Organizational ambidexterity for digital innovation: the approach of digital innovation labs. Acad Manag Glob Proc.

[CR72] Holotiuk F, Beimborn D (2019) Temporal ambidexterity: how digital innovation labs connect exploration and exploitation for digital innovation. In: Proceedings of the 40th international conference on information systems. ICIS, Munich, pp 1–17

[CR73] Hueske AK, Guenther E (2015). What hampers innovation? External stakeholders, the organization, groups and individuals: a systematic review of empirical barrier research. Manag Rev Q.

[CR74] Ireland RD, Kuratko DF, Covin JG (2003). Antecedents, elements, and consequences of corporate entrepreneurship strategy. Acad Manag Proc.

[CR75] Ireland RD, Webb JW (2007). Strategic entrepreneurship: creating competitive advantage through streams of innovation. Bus Horiz.

[CR76] Jansen JJP (2011) Corporate entrepreneurship: sensing and seizing opportunities for a prosperous research agenda. ERIM report series reference no. EIA-2011-046-STR

[CR77] Jansen JJP, Simsek Z, Cao Q (2012). Ambidexterity and performance in multiunit contexts: cross-level moderating effects of structural and resource attributes. Strateg Manag J.

[CR78] Jansen JJP, Tempelaar MP, van den Bosch FAJ, Volberda HW (2009). Structural differentiation and ambidexterity: the mediating role of integration mechanisms. Organ Sci.

[CR79] Jansen JJP, Van Den Bosch FAJ, Volberda HW (2006). Explorative innovation, exploitative innovation, and performance: effects of organizational antecedents and environmental moderators. Manag Sci.

[CR80] Jones G, Kraft A (2004). Corporate venturing: the origins of Unilever’s pregnancy test. Bus Hist.

[CR81] Kanbach D, Stubner S (2016) Corporate accelerators as recent form of startup engagement: the what, the why, and the how. J Appl Bus Res 32:1761–1776. 10.19030/jabr.v32i6.9822

[CR82] Katila R, Ahuja G (2002). Something old, something new: a longitudinal study of search behavior and new product introduction. Acad Manag J.

[CR83] Katkalo VS, Pitelis CN, Teece DJ (2010). Introduction: on the nature and scope of dynamic capabilities. Ind Corp Change.

[CR84] Kraus S, Breier M, Dasí-Rodríguez S (2020). The art of crafting a systematic literature review in entrepreneurship research. Int Entrep Manag J.

[CR85] Kupp M, Marval M, Borchers P (2017). Corporate accelerators: fostering innovation while bringing together startups and large firms. J Bus Strategy.

[CR87] Kuratko DF, Audretsch DB (2013). Clarifying the domains of corporate entrepreneurship. Int Entrep Manag J.

[CR88] Kuratko DF, Covin JG, Hornsby JS (2014). Why implementing corporate innovation is so difficult. Bus Horiz.

[CR89] Kuratko DF, Hornsby JS, Hayton J (2015). Corporate entrepreneurship: the innovative challenge for a new global economic reality. Small Bus Econ.

[CR90] Kuratko DF, Morris MH, Libecap G (2003). Corporate entrepreneurship: the dynamic strategy for 21st century organizations. Issues in entrepreneurship, advances in the study of entrepreneurship, innovation and economic growth.

[CR91] Lavie D, Rosenkopf L (2006). Balancing exploration and exploitation in alliance formation. Acad Manag J.

[CR92] Lavie D, Stettner U, Tushman ML (2010). Exploration and exploitation within and across organizations. Acad Manag Ann.

[CR93] Leten B, Van Dyck W (2012). Corporate venturing: strategies and success factors. Rev Bus Econ Lit.

[CR94] Madsen EL, Wall S, Zimmermann C, Klingebiel R, Lange D (2010). A dynamic capability framework–generic types of dynamic capabilities and their relationship to entrepreneurship. Strategic reconfigurations: building dynamic capabilities in rapid-innovation-based industries.

[CR95] Magnusson M, Martini A (2008). Dual organizational capabilities: from theory to practice—the next challenge for continuous innovation. Int J Technol Manag.

[CR96] Mahdjour S, Fischer S (2014). International corporate entrepreneurship with born global spin-along ventures—a cross-case analysis of telekom innovation laboratories’ venture portfolio. Int J Innov Manag.

[CR97] Majumdar SK (2000). Sluggish giants, sticky cultures, and dynamic capability transformation. J Bus Ventur.

[CR98] March JG (1991). Exploration and exploitation in organizational learning. Organ Sci.

[CR99] Marín-Idárraga DA, Hurtado-González JM, Cabello C (2016). The antecedents of exploitation-exploration and their relationship with innovation: a study of managers’ cognitive maps. Creat Innov Manag.

[CR100] Martin JA, Eisenhardt KM, Baum J, McGahan A (2004). Coping with decline in dynamic markets: corporate entrepreneurship and the recombinative organizational form. Business strategy over the industry lifecycle. Advances in strategic management.

[CR101] McGrath RG, Keil T, Tukiainen T (2006). Extracting value from corporate venturing. MIT Sloan Manag Rev.

[CR102] Michl T, Gold B, Picot A (2012). The spin-along approach: ambidextrous corporate venturing management. Int J Entrep Small Bus.

[CR103] Miles MP, Covin JG (2002). Exploring the practice of corporate venturing: Some common forms and their organizational implications. Entrep Theory Pract.

[CR104] Moschner S, Herstatt C (2017) All that glitters is not gold: how motives for open innovation collaboration with startups diverge from action in corporate accelerators. Working paper No. 102

[CR105] Moultrie J, Lewis M (2005). The organizational innovation laboratory. Creat Innov Manag.

[CR106] Nadkarni S, Prügl R (2021). Digital transformation: a review, synthesis and opportunities for future research. Manag Rev Q.

[CR107] Narayanan VK, Yang Y, Zahra SA (2009). Corporate venturing and value creation: a review and proposed framework. Res Policy.

[CR108] O’Connor GC, DeMartino R (2006). Organizing for radical innovation: an explorative study of the structural aspects of RI management systems in large established firms. J Prod Innov Manag.

[CR109] O’Hare J, Hansen P, Turner N, Dekoninck E (2008) Innovation hubs: why do these innovation superstars often die young? In: Marjanovic D, Storga M, Pavkovic N, Bojcetic N (eds) DS 48: proceedings DESIGN 2008, the 10th international design conference. Dubrovnik, Croatia, pp 971–978

[CR110] O’Reilly CA, Harreld JB, Tushman ML (2009). Organizational ambidexterity: IBM and emerging business opportunities. Calif Manag Rev.

[CR111] O’Reilly CA, Tushman ML (2004). The ambidextrous organization. Harv Bus Rev.

[CR112] O’Reilly CA, Tushman ML (2011). Organizational ambidexterity in action: how managers explore and exploit. Calif Manag Rev.

[CR113] O’Reilly CA, Tushman ML (2013). Organizational ambidexterity: past, present, and future. Acad Manag Perspect.

[CR114] O’Reilly CA, Tushman ML (2008). Ambidexterity as a dynamic capability: Resolving the innovator’s dilemma. Res Organ Behav.

[CR115] Papachroni A, Heracleous L, Paroutis S (2014). Organizational ambidexterity through the lens of paradox theory: building a novel research agenda. J Appl Behav Sci.

[CR116] Phan PH, Wright M, Ucbasaran D, Tan WL (2009). Corporate entrepreneurship: current research and future directions. J Bus Vent.

[CR117] Popadiuk S, Luz ARS, Kretschmer C (2018). Dynamic capabilities and ambidexterity: how are these concepts related?. Rev Adm Contemp.

[CR118] Raisch S (2008). Balanced structures: designing organizations for profitable growth. Long Range Plan.

[CR119] Raisch S, Birkinshaw J (2008). Organizational ambidexterity: antecedents, outcomes, and moderators. J Manag.

[CR120] Raisch S, Birkinshaw J, Probst G, Tushman M (2009). Organizational ambidexterity: balancing exploitation and exploration for sustained performance. Organ Sci.

[CR121] Raisch S, Tushman M (2016). Growing new corporate businesses: from initiation to graduation. Organ Sci.

[CR122] Rauch A, van Doorn R, Hulsink W (2014). A qualitative approach to evidence–based entrepreneurship: theoretical considerations and an example involving business clusters. Entrep Theory Pract.

[CR123] Reimsbach D, Hauschild B (2012). Corporate venturing: an extended typology. J Manag Control.

[CR124] Ricciardi F, Zardini A, Rossignoli C (2016). Organizational dynamism and adaptive business model innovation: the triple paradox configuration. J Bus Res.

[CR125] Rigtering J, Behrens MA (2021) The Effect of Corporate — Start-Up Collaborations on Corporate Entrepreneurship. Rev Manag Sci. 10.1007/s11846-021-00443-2

[CR126] Rossi M, Festa G, Papa A, Scorrano P (2019a) Corporate venture capitalists’ ambidexterity: myth or truth? IEEE Trans Eng Manag 1–12. 10.1109/TEM.2019.2903984

[CR127] Rossi M, Festa G, Fiano F, Giacobbe R (2019). To invest or to harvest? Corporate venture capital ambidexterity for exploiting/exploring innovation in technological business. Bus Proc Manag J.

[CR128] Sakhdari K (2016). Corporate entrepreneurship: a review and future research agenda. Tech Innov Manag R.

[CR129] Schilke O, Hu S, Helfat C (2018). Quo vadis, dynamic capabilities? A content-analytic review of the current state of knowledge and recommendations for future research. Acad Manag Ann.

[CR130] Schmitt A, Raisch S, Volberda HW (2018). Strategic renewal: past research, theoretical tensions and future challenges. Int J Manag Rev.

[CR131] Schoemaker PJH, Heaton S, Teece D (2018). Innovation, dynamic capabilities, and leadership. Calif Manag Rev.

[CR132] Schroll A, Mild A (2012). A critical review of empirical research on open innovation adoption. J Betriebswirtsch.

[CR133] Schuh G, Lau F, Herding J (2017). Description model for goals of corporate incubators. ISPIM Innov Symp.

[CR134] Shankar RK, Shepherd DA (2019). Accelerating strategic fit or venture emergence: different paths adopted by corporate accelerators. J Bus Vent.

[CR135] Sharma P, Chrisman JJ (1999). Toward a reconciliation of the definitional issues in the field of corporate entrepreneurship. Entrep Theory Pract.

[CR136] Shin BY, Cho KT (2020). The evolutionary model of corporate entrepreneurship: a case study of samsung creative-lab. Sustainability (switzerland).

[CR137] Simsek Z, Heavey C (2011). The mediating role of knowledge-based capital for corporate entrepreneurship effects on performance: a study of small- to medium-sized firms. Strat Entrep J.

[CR138] Simsek Z, Heavey C, Veiga JF, Souder D (2009). A typology for aligning organizational ambidexterity's conceptualizations, antecedents, and outcomes. J Manag Stud.

[CR139] Snehvrat S, Kumar A, Kumar R, Dutta S (2018). The state of ambidexterity research: a data mining approach. Int J Organ Anal.

[CR140] Stadler C, Rajwani T, Karaba F (2014). Solutions to the exploration/exploitation dilemma: networks as a new level of analysis. Int J Manag Rev.

[CR141] Taylor A, Helfat CE (2009). Organizational linkages for surviving technological change: complementary assets, middle management, and ambidexterity. Organ Sci.

[CR142] Teece D (2007). Explicating dynamic capabilities: the nature and microfoundations of (sustainable) enterprise performance. Strateg Manag J.

[CR143] Teece D (2014). The foundations of enterprise performance: dynamic and ordinary capabilities in an (economic) theory of firms. Acad Manag Perspect.

[CR144] Teece D (2018). Dynamic capabilities as (workable) management systems theory. J Manag Organ.

[CR145] Teece D, Pisano G (1994). The dynamic capabilities of firms: an introduction. Ind Corp Change.

[CR146] Teece D, Pisano G, Shuen A (1997). Dynamic capabilities and strategic management. Strateg Manag J.

[CR147] Teece DJ, Raspin PG, Cox DR (2020). Plotting strategy in a dynamic world. MIT Sloan Manag Rev.

[CR148] Tidd J, Taurins S (1999). Learn or leverage? Strategic diversification and organizational learning through corporate ventures. Creat Innov Manag.

[CR149] Tranfield D, Denyer D, Smart P (2003). Towards a methodology for developing evidence-informed management knowledge by means of systematic review. Br J Manag.

[CR150] Turner N, Swart J, Maylor H (2013). Mechanisms for managing ambidexterity: a review and research agenda. Int J Manag Rev.

[CR151] Tushman M, Smith WK, Wood RC, Westerman G, O’Reilly CA (2010). Organizational designs and innovation streams. Ind Corp Change.

[CR152] Tushman ML, O'Reilly CA (1996). Ambidextrous organizations: managing evolutionary and revolutionary change. Calif Manag Rev.

[CR153] Vanhaverbeke W, Peeters N (2005). Embracing innovation as strategy: corporate venturing, competence building and corporate strategy making. Creat Innov Manag.

[CR154] Vogel R, Güttel WH (2013). The dynamic capability view in strategic management: a bibliometric review. Int J Manag Rev.

[CR155] Wang CL, Ahmed PK (2007). Dynamic capabilities: a review and research agenda. Int J Manag Rev.

[CR156] Webster J, Watson RT (2002). Analyzing the past to prepare for the future: writing a literature review. MIS Quart.

[CR157] Weiblen T, Chesbrough HW (2015). Engaging with startups to enhance corporate innovation. Calif Manag Rev.

[CR158] Westerman G, McFarlan F, Iansiti M (2006). Organization design and effectiveness over the innovation life cycle. Organ Sci.

[CR159] Williams C, Lee SH (2009). Exploring the internal and external venturing of large RD-intensive firms. RD Manag.

[CR160] Winter SG (2003). Understanding dynamic capabilities. Strateg Manag J.

[CR161] Wolcott R, Lippitz M (2007). The four models of corporate entrepreneurship. MIT Sloan Manag Rev.

[CR162] Zahra SA (1991). Predictors and financial outcomes of corporate entrepreneurship: an explorative study. J Bus Ventur.

[CR163] Zollo M (2009). Superstitious learning with rare strategic decisions: theory and evidence from corporate acquisitions. Org Sci.

[CR164] Zollo M, Winter S (2002). Deliberate learning and the evolution of dynamic capabilities. Organ Sci.

