# Supplementary material for: Toward an integrated framework of corporate venturing for organizational ambidexterity as a dynamic capability
Source: Manag Rev Q. 2021 Jun 5;72(4):1129–70. doi: 10.1007/s11301-021-00223-y (PMC8179709; doi:10.1007/s11301-021-00223-y)
Supplement: Supplementary file 1 — Supplementary file1 (PDF 62 KB) [file 11301_2021_223_MOESM1_ESM.pdf]

Appendix I: Keyword list

Corporate Venturing (CV)		Dynamic capabilities (DC)		Organizational Ambidexterity (OA)	
Main keywords	***Specific***	***Main keywords***	***Specific***	***Main keywords***	***Specific***
Corporate ventur*	Open Innovation	Dynamic capabilities	sensing, seizing, transforming	Organizational ambidexterity	Structural ambidexterity
New venture development	Innovation lab	Dynamic capability	sensing, seizing reconfigur*	Organisational ambidexterity	Contextual ambidexterity
Corporate entrepreneurship	Digital lab	Organizational capabilities	sensing, Seizing, renew*	exploration exploitation	Sequential ambidexterity
	Innovation hub	Organisational capabilities		exploring exploiting	Ambidextrous leadership
	Corporate accelerator				opportunity-seeking advantage-seeking
	Venture Client				
	Corporate venture capital /CVC				
	Internal corporate venturing / ICV				
	New venture division/ NVD				
	Incubator, Incubation				
	Scale-up				
	Spin-out/-off/-along/-in				
