# Supplementary material for: Toward an integrated framework of corporate venturing for organizational ambidexterity as a dynamic capability
Source: Manag Rev Q. 2021 Jun 5;72(4):1129–70. doi: 10.1007/s11301-021-00223-y (PMC8179709; doi:10.1007/s11301-021-00223-y)
Supplement: Supplementary file 2 — Supplementary file2 (PDF 81 KB) [file 11301_2021_223_MOESM2_ESM.pdf]

Appendix II: Search strings

Topic	Search string	Results*
Round 1	***Including relevant specific keywords as possible synonyms for main keywords***	***4215***
DC-OA intersection	(Organizational Ambidexterity OR Organisational ambidexterity OR exploration exploitation OR Structural ambidexterity OR Contextual ambidexterity OR Sequential ambidexterity OR Ambidextrous leadership OR opportunity-seeking advantage-seeking) **AND** (Dynamic capabilit* OR organizational capabilities OR organisational capabilities OR sensing seizing transforming OR sensing seizing renconfig*, OR sensing seizing renew*)	1302
DC-CV intersection	(Corporate ventur* OR New venture development OR Corporate entrepreneurship OR Intrapreneurship OR Open Innovation OR Innovation lab OR Digital lab OR Innovation hub OR Corporate accelerator OR Venture Client OR Corporate venture capital OR Internal corporate venturing OR New venture division) **AND** (Dynamic capabilit* OR OR organizational capabilities OR organisational capabilities OR sensing seizing transforming OR sensing seizing renconfig*, OR sensing seizing renew*)	2386
CV-OA intersection	(Corporate ventur* OR New venture development OR Corporate entrepreneurship OR Intrapreneurship OR Open Innovation OR Innovation lab OR Digital lab OR Innovation hub OR Corporate accelerator OR Venture Client OR Corporate venture capital OR Internal corporate venturing OR New venture division) **AND** (Organizational Ambidexterity OR Organisational ambidexterity OR exploration exploitation OR Structural ambidexterity OR Contextual ambidexterity OR Sequential ambidexterity OR Ambidextrous leadership OR opportunity-seeking advantage-seeking)	527
Round 2	***Excluding relevant specific keywords as possible synonyms for main keywords***	***1427***
DC-OA intersection	(ambidexterity OR organisational ambidexterity OR organizational ambidexterity OR exploration exploitation OR exploring exploiting) **AND** (dynamic capabilities OR dynamic capability)	911
DC-CV intersection	(corporate ventur* OR corporate entrepreneurship OR new venture development) **AND** (dynamic capabilities OR dynamic capability)	581
CV-OA intersection	(ambidexterity OR organizational ambidexterity OR organisational ambidexterity OR exploration exploitation OR exploring exploiting) **AND** (corporate ventur* OR corporate entrepreneurship OR new venture development)	340

**EBSCOHost, March 15, 2020*
