# Supplementary material for: Toward an integrated framework of corporate venturing for organizational ambidexterity as a dynamic capability
Source: Manag Rev Q. 2021 Jun 5;72(4):1129–70. doi: 10.1007/s11301-021-00223-y (PMC8179709; doi:10.1007/s11301-021-00223-y)
Supplement: Supplementary file 3 — Supplementary file3 (PDF 74 KB) [file 11301_2021_223_MOESM3_ESM.pdf]

Appendix III: Coding scheme

**Source**	**Key concepts**	**Conceptualization**	**Integration**
(predefined)	***(predefined)***	***(derived from RQ and context)***	***(derived from analysis)***
Year	DC background *(summary)*	OA - DC intersection *(specific findings)*	OA logic *(trade-off view - paradox view)*
Focus category *(DC, OA, CV/CE)*	OA background *(summary)*	CV - DC intersection *(specific findings)*	DC logic *(structures/processes - individual behaviour)*
Method *(review, conceptual, qualitative, quantitative)*	CE/CV background *(summary)*	CV - OA intersection *(specific findings)*	CV setup *(separated, integrated, semi-structured, contextual)*
Research Design *(specifications)*			CV descriptions *(summary)*
Key findings *(summary)*			
