# Supplementary material for: Toward an integrated framework of corporate venturing for organizational ambidexterity as a dynamic capability
Source: Manag Rev Q. 2021 Jun 5;72(4):1129–70. doi: 10.1007/s11301-021-00223-y (PMC8179709; doi:10.1007/s11301-021-00223-y)
Supplement: Supplementary file 4 — Supplementary file4 (PDF 82 KB) [file 11301_2021_223_MOESM4_ESM.pdf]

Appendix IV: Concept matrix DC-OA-CV intersections (analysis)

Source	DC background	OA background	CV/CE background	OA-DC intersection	CV-DC intersection	CV-OA intersection
Adner & Helfat, 2003	x					
Akwei, Peppard & Hughes, 2008	x					
Alänge & Steiber, 2018						x
Almahendra & Ambs, 2014		x				
Ambrosini & Bowman, 2009	x				x	
Anand, Oriani & Vassolo, 2010					x	
Andriopoulos & Lewis, 2009		x				x
Anil et al., 2006		x				x
Arend & Bromiley, 2009	x				x	
Baden-Fuller & Volberda, 1997						x
Barney, 1991	x					
Barney, Wright & Kretchen, 2001	x					
Barreto, 2010	x					
Battistini, Hacklin & Baschera, 2013			x			x
Benner & Tuschman, 2003		x		x		x
Bierwerth, Schwens & Isidor, 2015			x			
Birkinshaw & Gupta, 2013						x
Birkinshaw, Batenburg & Murray, 2002			x			x
Birkinshaw, Zimmermann & Raisch, 2016				x		x
Blindenbach-Driessen & Van den Ende, 2014						x

Source	DC background	OA background	CV/CE background	OA-DC intersection	CV-DC intersection	CV-OA intersection
Breznik & Hisrich, 2014					x	
Burgelman & Välikangas, 2005			x			x
Burgelman, 1983			x		x	x
Burgelman, 1984a			x			
Burgelman, 1984b			x			x
Burgelman, 1985			x			x
Burgers & Jansen, 2008		x	x			x
Callegari & Rai, 2021		x				
Campbell et al., 2003						x
Cantarello, Martini & Nosella, 2012						x
Castiaux, 2012	x					
Cavusgil, Seggie & Talay, 2007	x					
Chen, 2017		x				x
Chesbrough, 2000						x
Chesbrough, 2002						x
Christensen & Bower, 1996						x
Collis, 1994	x					
Corbett et al., 2013			x			
Covin & Miles, 1999			x			
Covin & Miles, 2007			x		x	x
Covin et al., 2015						x
Crockett, McGee & Payne, 2013						x
Du & Chen, 2018	x	x				x
Dushnitsky & Birkinshaw, 2014			x			x

Source	DC background	OA background	CV/CE background	OA-DC intersection	CV-DC intersection	CV-OA intersection
Easterby-Smith, Lyles & Peteraf, 2009	x					
Eisenhardt & Martin, 2000	x			x	x	
Eisenhardt, Furr & Bingham, 2010		x				x
Enkel & Sagmeister, 2020					x	x
Eriksson, 2014	x				x	
Farjoun, 2010		x				x
Garrett & Neubaum, 2013			x			x
Gassmann & Becker, 2006					x	x
Gibson & Birkinshaw, 2004		x				x
Gilbert, 2006						x
Gimmy et al., 2017			x			x
Gonthier & Chirita, 2019					x	x
Gupta, Smith & Shalley, 2006		x				x
Gutmann et al., 2020						x
Gutmann, 2019			x			x
Gutmann, Kanbach & Seltmann, 2019						x
Helfat & Peteraf, 2009	x					
Helfat & Winters, 2011	x				x	
Heracleous et al., 2017						x
Hill & Birkinshaw, 2005			x			x
Hill & Birkinshaw, 2006						x
Hill & Birkinshaw, 2008			x			x
Hill & Birkinshaw, 2012		x	x			x

Source	DC background	OA background	CV/CE background	OA-DC intersection	CV-DC intersection	CV-OA intersection
Hill & Georgulas, 2016			x		x	x
Holotiuk & Beimborm, 2018						x
Ireland & Webb, 2007		x	x			x
Ireland, Covin & Kuratko, 2008			x			
Jansen et al., 2009				x		x
Jansen, Simsek & Cao, 2012		x				x
Jansen, Van den Bosch & Volberda, 2006			x			x
Jones & Kraft, 2004			x			x
Jurksiene & Pundziene, 2016				x		
Kanbach & Stubner, 2016						x
Kanter, 1985		x				x
Katkalo, Pitelis & Teece, 2010	x			x	x	
Keil et al., 2008						x
Keil, 2001			x			x
Kodama, 2017				x		
Koza & Lewin, 2000			x			x
Kruft & Kock, 2019			x			x
Kupp, Marval & Borchers, 2017						x
Kuratko & Audtretsch, 2013			x			
Kuratko & Morris, 2003	x				x	x
Kuratko, 2010			x			x
Kuratko, Covin & Garrett, 2009			x			x
Kuratko, Covin & Hornsby, 2014			x			x

Source	DC background	OA background	CV/CE background	OA-DC intersection	CV-DC intersection	CV-OA intersection
Kuratko, Hornsby & Hayton, 2015			x			
Lavie & Rosenkopf, 2006		x				x
Lavie, Stettner & Tushman		x				x
Lawson & Samson, 2001					x	
Leiblein, 2011	x					
Leten & Dyck, 2012		x	x			x
Lubatkin et al., 2006						x
Madsen, 2010	x	x		x	x	
Magnusson & Martini, 2012			x			
Mahdjour & Fischer, 2014						x
Majumdar, 2000	x					
March, 1991		x				x
Marín-Idárraga, Hurtado-González, & Cabello (2016)		x		x		x
Martin & Eisenhardt, 2004					x	x
Martini et al., 2013						x
Mathias, 2014		x				
McGrath, Keil & Tukainen, 2006						x
Michl, Gold & Picot, 2012			x			x
Miles & Covin, 2002						x
Moreno et al., 2015						x
Moschner & Herstatt, 2018			x			x
Narayanan, Yang & Zahra, 2009			x			x
O'Reilly & Tushman, 2004		x				

Source	DC background	OA background	CV/CE background	OA-DC intersection	CV-DC intersection	CV-OA intersection
O'Reilly & Tushman, 2013			x	x		x
O’Cass, Heirato & Ngo, 2014						x
O’Connor & DeMartino, 2006						x
O’Hare et al., 2008						x
O’Reilly & Tushman, 1996		x				x
O’Reilly & Tushman, 2008	x	x		x		x
O’Reilly & Tushman, 2011	x	x		x		x
O’Reilly, Harreld & Tushman, 2009			x	x		x
Papachroni, Heracleous & Paroutis, 2014						x
Pavlou & El Sawy, 2011				x	x	
Pisano, 2017	x					
Pitelis, 2009		x				
Popadiuk, Luz & Kretschmer, 2018				x		
Raisch & Birkinshaw, 2008		x		x		
Raisch & Tushman, 2016			x			x
Raisch et al., 2009		x		x		x
Raisch, 2008		x				x
Reimbach & Hauschild, 2012			x			x
Rigtering & Behrens, 2021					x	
Rohrbeck, Döhler & Arnold, 2009						x
Rossi et al., 2019						x
Sakhdari, 2016			x			
Sarkees & Hulland, 2009						x

Source	DC background	OA background	CV/CE background	OA-DC intersection	CV-DC intersection	CV-OA intersection
Schildt, Maula & Keil, 2005						x
Schilke, Hu & Helfat, 2018	x					
Schuh, Lau & Herding, 2017		x				x
Sharma & Chrisman, 1999			x		x	
Shaw, O'Loughlin & McFadzean, 2005			x			
Shin & Cho, 2020						x
Shoemaker, Heaton & Teece, 2018	x					
Simsek et al., 2009		x				x
Snehvrat et al., 2018		x		x		x
Taródy, 2016		x				x
Taylor & Helfat, 2009						x
Teece & Augier, 2009	x				x	
Teece & Pisano, 1994	x			x		
Teece, 2007	x			x	x	
Teece, 2014	x					
Teece, 2018	x					
Teece, 2020	x					
Teece, Pisano & Shuen, 1997	x					
Thornhill & Amit, 2001						x
Tidd & Tauriin, 1999						x
Titus, House & Covin, 2017						x
Turner, Swart & Maylor, 2013		x				
Tushman et al., 2010						x
Vanhaverbeke & Peeters, 2005					x	x

Source	DC background	OA background	CV/CE background	OA-DC intersection	CV-DC intersection	CV-OA intersection
Vogel & Güttel, 2012				x		
Wang & Ahmed, 2007				x		
Weiblein & Chesbrough, 2015			x			x
Westerman & McFarlan, 2006						x
Williams & Lee, 2009					x	x
Winter, 2003	x				x	
Wolcott & Lippitz, 2007			x			x
Wulf, Stubner & Blarr, 2010						x
Yang, Cul & Pan, 2016						x
Zahra & Sapienza, 2006					x	
Zahra, 1991			x		x	x
Zollo & Winter, 2002	x			x		
