# Supplementary material for: Toward an integrated framework of corporate venturing for organizational ambidexterity as a dynamic capability
Source: Manag Rev Q. 2021 Jun 5;72(4):1129–70. doi: 10.1007/s11301-021-00223-y (PMC8179709; doi:10.1007/s11301-021-00223-y)
Supplement: Supplementary file 5 — Supplementary file5 (PDF 83 KB) [file 11301_2021_223_MOESM5_ESM.pdf]

Appendix V: Concept matrix CV for OA as a DC (results)

Source	Organizational ambidexterity logic (from analysis)		Dynamic capability logic (from analysis)		CV setup
	Trade-off view (system level)	*Paradox view (unit/individual level)*	*Organizational structures/ processes*	*Individual behaviour / routines*	*separated, separated-integrated, contextual, interlinked*
Alänge et al., 2018		x	x		interlinked-ambidextrous
Andriopoulos & Lewis, 2009		x		x	contextual-ambidextrous
Baden-Fuller & Volberda, 1997	x		x		separated
Birkinshaw, Zimmermann & Raisch, 2016	x			x	separated-integrated
Burgelman & Valikangas, 2005		x	x		interlinked-ambidextrous
Burgelman, 1985		x	x		interlinked-ambidextrous
Cantarello, Martini, & Nosella, 2012		x	x		interlinked-ambidextrous
Chesbrough, 2000		x	x		interlinked-ambidextrous
Chesbrough, 2002		x	x		interlinked-ambidextrous
Christensen & Bower, 1996	x	…	x		separated
Du & Chen, 2018		x	x		interlinked-ambidextrous
Enkel & Sagmeister, 2020		x	x		interlinked-ambidextrous
Galunic & Eisenhardt, 2001 (in: Eisenhardt & Martin, 2004)		x	x		interlinked-ambidextrous
Gassmann & Becker, 2004		x	x		interlinked-ambidextrous
Gilbert, 2006	x			x	separated-integrated
Gimmy et al., 2017		x	x		interlinked-ambidextrous
Gutmann et al., 2020		x	x		interlinked-ambidextrous
Gutmann, Kanbach & Seltman, 2019		x	x		interlinked-ambidextrous
Heracleous et al., 2017	x		x		separated
Holotiuk & Beimborm, 2018		x	x		interlinked-ambidextrous
Jones & Kraft, 2004	x		x		separated
Kanbach & Stubner, 2016	x			x	separated-integrated
Kupp, Marval & Borchers, 2017	x			x	separated-integrated
Leten & Dyck, 2015		x	x		interlinked-ambidextrous
Mahdjour & Fischer, 2014	x			x	separated-integrated

Source	Organizational ambidexterity logic (from analysis)		Dynamic capability logic (from analysis)		CV setup
	Trade-off view (system level)	*Paradox view (unit/individual level)*	*Organizational structures/ processes*	*Individual behaviour / routines*	*separated, separated-integrated, contextual, interlinked*
McGrath, Keil & Tikiainen, 2006		x	x		interlinked-ambidextrous
Michl, Gold, & Picot, 2012		x	x		interlinked-ambidextrous
Miles & Covin, 2002		x		x	contextual-ambidextrous
Miles & Covin, 2007	x		x		separated
Miles & Covin, 2007		x	x		interlinked-ambidextrous
Miles & Covin, 2007		x		x	contextual-ambidextrous
Moschner & Herstatt, 2018	x		x		separated
O'Connor & DeMartino, 2006		x	x		interlinked-ambidextrous
O'Hare et al., 2008		x	x		interlinked-ambidextrous
O'Reilly & Tushman, 2004	x			x	separated-integrated
O'Reilly & Tushman, 2011	x			x	separated-integrated
O'Reilly, Harreld & Tushman, 2009	x			x	separated-integrated
Raisch & Tushman, 2016		x	x		interlinked-ambidextrous
Raisch, 2008		x	x		interlinked-ambidextrous
Shin & Cho, 2020		x	x		interlinked-ambidextrous
Taylor & Helfat, 2009		x	x		interlinked-ambidextrous
Tidd, J., & Taurins, S., 1999		x	x		interlinked-ambidextrous
Tushman & O'Reilly, 1996	x			x	separated-integrated
Tushman et al., 2010	x		x		separated
Tushman et al., 2010	x			x	separated-integrated
Van Haverbeke & Peeters, 2005		x	x		interlinked-ambidextrous
Weiblen & Chesbrough, 2015		x	x		interlinked-ambidextrous
Westerman & McFarlan, 2006		x	x		interlinked-ambidextrous
Wolcott & Lippitz, 2007		x		x	contextual-ambidextrous
