# Supplementary material for: Toward an integrated framework of corporate venturing for organizational ambidexterity as a dynamic capability
Source: Manag Rev Q. 2021 Jun 5;72(4):1129–70. doi: 10.1007/s11301-021-00223-y (PMC8179709; doi:10.1007/s11301-021-00223-y)
Supplement: Supplementary file 6 — Supplementary file6 (PDF 150 KB) [file 11301_2021_223_MOESM6_ESM.pdf]

Appendix VI: Synthesis of selected empirical studies in the integrated framework of CV for OA as DC

Source	Methodology	Category-defining insights
Separated corporate venturing		
Christensen & Bower, 1996	Multiple case study of 3 disrutive corporate ventures from established companies in the disk drive industry	Two CV cases can be defined as separated from the core organization, and one as fully integrated<br>Identifies the two cases with independent CV organizations as successful<br>Describes one case with an integrated CV organization to require extreme managerial effort<br>Separate, independent CV can be found more successful with higher business growth (for the new venture)
Baden-Fuller & Volberda, 1997	Multiple case study, including Xerox, IBM, Eastman Kodak, Philips	CV cases of Xerox, IBM, Kodak, and Philips can be defined as separated from the core business<br>Describes successful exploration, but high difficulty in transferring new capabilities into the core business<br>Identifies lack of communication channels and common mental frames as barriers to exploitation at IBM<br>Separated CV can only be found medium successful due to limited exploitation
Jones & Kraft, 2004	Historical single case study of Unilever's Unipath CV unit	CV case of Unipath unit can be defined as organizationally and culturally separated<br>Identifies political problems due to 'orphaning' of the ventures<br>Describes growing tensions despite remarkable success of Unipath with profitable medical business, due to low impact of only 0.3 per cent on the overall core business and the distinct philosophy and culture<br>Separated CV can be found unsuccessful as it did not survive a new focus on the core business
Miles & Covin, 2007	Multiple case study of CV units in 15 UK and Swedish firms with specific examples of C&W and Telia	CV cases of C&W and Telia can be defined as autonomous units with purely financially driven goals<br>Confirms successful exploration, but barriers to exploitation from Baden-Fuller and Volberda (1997)<br>Identifies limited innovation transfer, or pursuit of strategically irrelevant opportunities due to missing link between CV and business strategy<br>Separated CV can be found unsuccessful with units shut down, failing to contribute to the operating performance, or to augment the corporations scope of operations with significant innovation / new business
Tushman et al., 2010	Longitudinal, multiple case study of 13 business units and 22 innovations with specific example of USAToday	CV case on new online business USAToday.com can be defined as separated 'Spin-out' CV without any linking mechanisms (at first)<br>Identifies resource challenges for further growth due to failure of senior management support<br>Separated CV can be found unsuccessful due to hindered growth (and following change of setup)

Source	Methodology	Category-defining insights
Heracleous et al., 2017	Historical single case study of Xerox PARC	CV case can be defined as geographically, culturally and organizational separated<br>Identifies successful exploration, but poor exploitation, lacking any link to the core business<br>Separated CV can be found unsuccessful as ‘orphan‘ technologies were either terminated or commercialized outside of the organization
Moschner & Herstatt, 2018	Multiple case study of 10 corporate accelerators	CV cases of corporate accelerators can be defined as separated with complementary assets not being used collaboratively<br>Identifies failure in exploiting opportunities due to lack of interest from core organization to use assets collaboratively with the startup ventures of the corporate accelerator<br>Describes ‘entrepreneurial washing’ with the use of open innovation rather as a symbolic marketing tool than actual collaboration<br>Separated CV can be found unsuccessful due to failure of exploitation with the core business
Separated-integrated corporate venturing		
Tushman & O’Reilly, 1996	Multiple case study of HP, J&J and ABB	CV cases of Hewlett-Packard, Johnson & Johnson, and ABB can be defined as autonomous units, integrated on the corporate level<br>Describe successful evolutionary & revolutionary change in all cases with incremental innovation in mature markets and discontinuous innovation in emerging markets & technologies<br>Identifies OA based autonomous groups with leadership managing both alignment and revolution as success factor<br>Describes managers to require organizational and management skills for both exploring and exploiting at the same time<br>Separated-integrated CV can be found successful for overcoming the tensions between the current and future business
O’Reilly & Tushman, 2004	Multiple case study of 35 attempts to launch breakthrough innovations in 15 business units across nine different industries	15 CV cases can be defined as separated, structurally independent units with own processes, structures, and cultures, but integrated into the existing senior management hierarchy<br>Identifies tight coordination at the management level as success factor to enable the sharing of important resources such as cash, talent, expertise, customers, etc.<br>Identifies organizational separation as success factor to shield new units from ‘business as usual’, and established units from distractions of launching a new business<br>Separated-integrated CV can be found successful to launch breakthrough innovations

Source	Methodology	Category-defining insights
Gilbert, 2006	Longitudinal, multilevel single case study of a newspaper organization	CV case on new digital publishing in a newspaper organization can be defined as separated subunits, integrated within individuals on the corporate level<br>Describes coexistence of competing frames of exploration and exploitation based on organizationally differentiated subunits with an increased burden on senior teams to manage the inconsistencies across subunits<br>Identifies lack of direct operating responsibilities to allow engagement in different frames at the corporate level<br>Separated-integrated CV can be found successful with frame integration occurring within individuals at the corporate level
O'Reilly, Harreld & Tushman, 2009	Single case study of IBM EBO	CV case of the IBM Emerging Business Organization can be defined as organizationally separated, but integrated by the senior management team<br>Identifies variation, selection and retention process to be regulated across separated units<br>Identifies success factor of the senior management teams ability to regulate the variation - selection-retention process in a way that maintains the ecological fitness of the organization with its environment<br>Separated-integrated CV can be found successful in helping an organization to adapt to new markets due to the integration at the senior management level
Tushman et al., 2010	Longitudinal, multiple case study of 13 business units and 22 innovations with specific example of USAToday	CV case on new approach of online USAToday.com business can be defined as organizationally separated, but integrated through a new senior management team<br>Describes integration on the corporate level with establishment of editorial meetings with senior management team of the core business, and new incentives of the senior management to be based on both core and new business<br>Identifies organizational ambidexterity to be based on differentiated organization with with targeted editorial linkages and strong senior team integration<br>Separated-integrated CV can be found successful depending on the senior team's ability to manage contradictory internal architectures

Source	Methodology	Category-defining insights
O'Reilly & Tushman, 2011	Multiple case study of senior managers in 15 firms that were attempting to manage both exploratory and exploitative units	CV cases with a clear strategic intent to pursue an explorative venture can be defined to be separated organizationally, and integrated by the senior management team<br>Describes senior-level integration to ensure that resources are allocated, e.g. through the EBO process at IBM, a portfolio approach at Middleware, or a Boards and Councils process at Cisco<br>Separated-integrated CV can be found successful due to physical separation from the exploit parts of the business, and senior management integration with a clear, identifiable leader and forum to resolve tensions and allocate resources.
Mahdjour & Fischer, 2014	Multiple case study of Deutsche Telekom Innovation laboratories spin-along program	CV case of T-Labs spin-along program can be defined as separated in new business organizations with integration by the corporate level through a close linkage and monitoring after market launch<br>Describes spin-out of ideas into separate ventures, which keep a close linkage and monitoring after market launch through regular evaluation and decision to re-integrate, keep at distance or reduce ownership of shares by the management team<br>Separated-integrated CV can be found successful when kept at distance for a longer periods of time with continuous linkage to the core business through the management team
Birkinshaw, Zimmermann & Raisch, 2016	Multiple case study of Nestlé SA, GlaxoSmithKline (GSK), and the BMW Group (BMW)	CV case of Nestlé Nutrition can be defined to be structurally separated, but integrated with a unifying vision and aligned goals on the senior management level<br>Identifies sensing and seizing capabilities to be created in different units, while the unifying vision on the corporate level aligns them as a whole<br>Separated-integrated CV can be found successfully serving the complementary objectives
Kanbach & Stubner, 2016	Multiple case study of 13 corporate accelerators in Germany	CV cases of corporate accelerators can be defined as being separated on the operational level, but integrated on the strategic level as the focus on either exploration or exploitation<br>Describes that although most programs follow exploration and exploitation logic simultaneously, usually one form of logic dominates<br>Finds exploration-oriented programs successful to focus on understanding of market trends and developments, as well as identifying the responsible entrepreneurial startups<br>Separated-integrated CV can be found successful to focus on the use of existing parent company competencies to improve startup development

Source	Methodology	Category-defining insights
Kupp, Marval & Borchers, 2017	Single Case study of Deutsche Telekom hub:raum CV unit	CV case of hub:raum from Deutsche Telekom can be defined as separated unit, integrated by the support of the CEO and high-ranking sponsors<br>Describes involvement of CEO René Obermann in the beginning to setup the CV unit, the following CEO Tim Höttges visibly supporting startup investments and partnerships to integrate innovation and gradually transform how Deutsche Telekom innovates, and high-ranking managers as sponsors to mentor startup teams<br>Separated-integrated CV can be found successful for turning the corporate culture into an increasingly open breeding ground for connecting internal and external innovation
Enkel & Sagmeister, 2020	Multiple case study of external corporate venturing	Multiple case study of external CV modes can be categorized as separated-integrated due to focus on external exploration and exploitation and required linking of different CV modes for DCs<br>Describes how firms use CV to identify and exploit startup's competencies<br>Identifies application and linking of different CV modes as requirement to build / contribute to dynamic capabilities<br>Full potential of separate-integrated CV modes not reached by all firms, as linking and portfolio of difference (separate) CV modes are not always applied
Contextual-ambidextrous corporate venturing		
Miles & Covin, 2002	Multiple case study of CV programs in 11 firms	CV cases of 3M and P&G can be defined as contextually ambidextrous based on employees exploring and exploiting new ideas within the corporate structure<br>Describes how employees of 3M can dedicate 15% of their time on (unstructured) exploration, and P&G encourages employees to act as intrapreneurs for (structured) exploration<br>Contextual-ambidextrous CV can be found successful to constantly refresh the corporations product mix and re-energizing an innovative culture

Source	Methodology	Category-defining insights
Miles & Covin, 2007	Multiple case study of CV units of 15 UK and Swedish firms with specific examples of 3M, Skandia AB and P&G	CV cases of 3M, Skandia AB and Ericsson can be defined as contextually ambidextrous, enabling the individual balance of exploration and exploitation<br>Describes an innovation center at 3M to connect multiple stakeholders, and a two-way communication channel between management and corporate entrepreneurs at Skandia Ab and Ericsson as context for the individual balance of exploration and exploitation across the members of the organization<br>Identifies the success factor of regular interaction across stakeholders to identify and pursuit entrepreneurial opportunities at 3M<br>Identifies the success factor of direct links between top management and lower-level corporate entrepreneurs, as well as a role for all organizational members in tightly linked strategy formation and entrepreneurial processes<br>Contextual-ambidextrous CV can be found successful as many ventures emerged from the programs, such as 3M's transdermal drug delivery system
Wolcott & Lippitz, 2007	Multiple case study of corporate entrepreneurship at nearly 30 global companies	CV case of Google can be defined as contextually ambidextrous based on slack-time to enable exploration for employees in exploitative business units<br>Describes "enabler" CV model that supports employees across the organization to develop new concepts with adequate support<br>Identifies success factor of dedicated resources and processes, but without any formal organizational ownership to enable corporate entrepreneurs the pursuit of opportunities on their own (insofar they fit the organization's strategic frame)<br>Identifies success factors of clear criteria for selecting opportunities, application guidelines for funding, decision-making transparency, recruitment and retention of entrepreneurially-minded employees and active support from senior management in most evolved versions of the enabler model<br>Describes Google as the poster child of the enabler model in which employees can spend 20% of their time to explore and exploit new opportunities<br>Contextual-ambidextrous CV can be found successful depending on the identified support mechanisms

Source	Methodology	Category-defining insights
Andriopoulos & Lewis, 2009	Multiple case study of 5 leading product design firms	CV cases can be defined as contextually ambidextrous with knowledge workers choosing themselves when and how to focus their skills on exploration or exploitation
		Describes multilevel approach in which managing innovation paradox becomes the responsibility of actors throughout the firm
		Identifies success factors of executives to set the context, providing leadership and allocating resource for projects, directors and project leaders to guide specific projects, ensuring adherence and encouraging improvisation, and knowledge workers choosing their work mode to support specific projects.
		Contextual-ambidextrous CV can be found successful as ambidexterity becomes pervasive in a virtuous cycle reinforced by the multilevel efforts
Du & Chen, 2018	Comparative case study of Tencent and Alibaba	CV cases of both Alibaba and Tencent can be defined as contextually ambidextrous (among others), building on innovations evolving from the employees
		Describes need for exploitation with execution and efficiency, while at the same time encouraging grassroots initiatives to improve consumer experience at Alibaba
		Describes spontaneous bottom-up innovations based on a strong sense of engineering culture with a spirit of multiple iteration and continuous improvement that pushes Tencent forward
		Contextual-ambidextrous CV can be found partly successful with promising products coming from first-line staff members and not managers, but hardly replicable as evolving opportunities can not be planned, and it requires a specific organizational culture and skillset
Interlinked-ambidextrous corporate venturing		
Burgelman, 1985	Single case study of a new venture division	CV case of the new venture division can be defined as interlinked-ambidextrous based on integrative mechanisms involving middle managers
		Describes impracticality of decoupling new ventures completely from the core business and integrative mechanisms such as steering committees from middle managers to overcome this
		Identifies success factor of middle managers acting as crucial linking and technology transfer mechanisms in the corporation
		Interlinked-ambidextrous CV can be found successful for better articulating a strategy for new business fields based on new combinations of existing capabilities and skills (which may again enhance the core business)

Source	Methodology	Category-defining insights
Tidd, J., & Taurins, S., 1999	Multiple case study of 15 UK-based firms	CV case of ICON from Imperial College can be defined as interlinked-ambidextrous in which relevant processes are shared across the new venture unit and at ground level<br>Describes responsibilities of CV unit for specific processes such as marketing and contracting, with other processes such as supply shared with the core business<br>Describes how the CV explores new opportunities both internally and externally before exploiting them with interlinked organizational processes and structures<br>Interlinked-ambidextrous CV can be found successful for learning (exploration) and leveraging (exploitation) across the organization
Chesbrough, 2000	Single case study of Lucent NVG	CV case of the Lucent new venture group can be identified as interlinked-ambidextrous based on operational links to the core business<br>Describes hybrid vehicle connecting working closely with the business units to exploit existing resources, while keeping an independent ability to explore new opportunities and business models and take them to market<br>Interlinked-ambidextrous CV can be found successful for balancing aspects of private venture capital with other aspects of the corporate mission as a hybrid model
Galunic & Eisenhardt, 2001<br>*(in: Eisenhardt & Martin, 2004)*	Inductive single case study of „OMNI“	Case on ‚OMNI‘ firm can be defined as interlinked-ambidextrous with ‚'loose-coupling' of different business and units and the corporate level<br>Describes neither disconnected, nor integrated, but rather aligned 'loose-coupling' of different units and divisions through shared management practices<br>Identifies success factor of alignment by simple standardized coordination mechanisms on the business and corporate level, e.g. shared common management practices, product development processes, human resource practices, and financial systems<br>Interlinked-ambidextrous CV can be found successful for facilitating movement of individuals and projects among the divisions and subsequent resource combinations

Source	Methodology	Category-defining insights
Chesbrough, 2002	Multiple case study of CVC	CV cases of Microsoft, Lucent, Cisco, Intel, and Panasonic can be defined as interlinked-ambidextrous, showing strong links of new ventures (start-ups) with the core business<br>Describes that start-ups build strong links to the investing company to leverage its existing assets such as manufacturing, distribution, technology, business practices or brand<br>Describes that start-ups may adopt the investing company's business practices to sell, build, or service their products<br>Interlinked-ambidextrous CV can be found successful in the CVC investment model of 'driving investments' with a strategic rationale and tight links between a start-up and the operations of the investing company
Gassmann & Becker, 2004	Multiple case study of 22 corporate incubators	CV cases of corporate incubators can be defined as interlinked-ambidextrous depending on resource-flow between parent and CV unit<br>Describes dependency on a continuous resource flow from parent to corporate incubator<br>Identifies success factors of deliberate alignment between CV and parent company's goals, as well as strong networking support<br>Identifies potential problems of exchange and communication of the separation between incubator and parent is too great<br>Interlinked-ambidextrous CV can be found successful for supporting strategic projects that could ensure the long-term existence of the company
Burgelman & Valikangas, 2005	Multiple case study of Intel and UPM	CV cases of Intel and UPM can be defined as interlinked-ambidextrous based on their need for organizational links<br>Describes that CV must become a core growth vehicle that can involve every business and all senior executives of the organization<br>Describes that links between new ventures and mainstream businesses must be explored and optimally exploited, instead of 'putting them away' in a separate unit<br>Identifies success factor of continuous integration of CV in the strategy-making process rather than an insurance policy against declining core business<br>Interlinked-ambidextrous CV can be found successful as integrated and continuous core growth vehicle, based on a broader range of structural arrangements that can address the interdependence between new and existing business

Source	Methodology	Category-defining insights
Van Haverbeke & Peeters, 2005	Single case study of DSM	CV case of the dutch chemical manufacturer DSM can be defined as interlinked-ambidextrous based on organizational linkages<br>Describes different organizational routines and procedures such as a 'Corporate Strategic Dialogues' to assure that new ideas from different parts of the company could be discussed as potential building blocks for the future corporate strategy<br>Interlinked-ambidextrous CV can be found successful as a practice that forces the firm to adapt its competencies over time and as a catalyst to recognize new strategic options
McGrath, Keil & Tikiainen, 2006	Single case study of Nokia NVO	CV case on Nokia's new venture organization can be defined as interlinked-ambidextrous, set up like a service business within the organization<br>Describes NVO to be structured like a service business to support exploration and exploitation throughout the organization with projects (and people) moving between the NVO and the core business<br>Describes NVO as complementary to the core business, doing things that the preoccupied management team of a core business could not do at all or could only do slowly<br>Interlinked-ambidextrous CV can be found successful especially in creating important new technologies, capabilities and products that help the core business adapt to change, besides successful new businesses
Westerman & McFarlan, 2006	Paired case studies of 4 innovation units	CV cases such as Charles Schwab and Walgreens can be defined as interlinked-ambidextrous based on three different hybrid designs<br>Describes that contrary to many theoretical recommendations, no CV approach is purely autonomous or integrated, but always structurally linked<br>Identifies no optimal design, but three viable adaption modes: separate-early, integrate-early, and wait-then-transform<br>Describes informal ties between old and new business at Charles Schwab combined with a disciplined approach to reduce unnecessary variation<br>Describes small differentiated innovating units linked with many existing units, often through reciprocal interdependencies at Walgreens<br>Interlinked-ambidextrous CV can be found successful, depending on the requirement of strong formal and informal integration mechanisms

Source	Methodology	Category-defining insights
O'Connor & DeMartino, 2006	Longitudinal study of radical innovation in 12 established firms	CV cases on radical innovation can be defined as interlinked-contextual based on different identified models with interface mechanisms to the mainstream organization<br>Describes access to assets and resources of the organization as potential competitive advantage over start-ups<br>Identifies interface mechanisms as success factor to allow allow the assimilation of radical innovation into the mainstream organization that can be seen indifferent types such as idea generator, incubator, sequential model, CV unit, R&D management system, self-similar model, and mirrored model<br>Interlinked-ambidextrous CV can be found successful to gain a competitive advantage over start-ups by leveraging and stretching current competencies and simultaneously building new ones
Miles & Covin, 2007	Multiple case study of CV units in 15 UK and Swedish firms with specific examples of Ericsson, 3M, Skandia, Generics group	CV cases on Ericsson, Intel, 3M, Skandia, Generics Group, and others can be defined as interlinked-ambidextrous, linking CV and business strategy in a relationship of 'reciprocal causality', or 'CV-as-a-strategy'<br>Describes models of reciprocal causality and CV-as-a-strategy to leverage existing organizational capabilities as interlinked units<br>Describes strategically treatment of entrepreneurial initiatives as mechanisms for deepening and leveraging the core competencies of the firm<br>Identifies success factor of the venture leveraging organizational knowledge or capabilities involving current markets or technologies (on anecdotal evidence)<br>Interlinked-ambidextrous CV can be found successful as the potentially most effective relationship between CV and business strategy, often effectively employed as a learning tool
Raisch, 2008	Inductive multiple case study of six leading Central European companies	Some CV cases can be defined as interlinked-ambidextrous with 'nurturing' and 'sharing' integration mechanisms<br>Describes simultaneous pursuit of exploitation and exploration within a unit despite the unit's primary orientation towards exploration<br>Interlinked-ambidextrous CV can be found successful depending on a well-balanced integration relying on integration mechanisms

Source	Methodology	Category-defining insights
O'Hare et al., 2008	Multiple case study of 6 Innovation Hubs	Some CV cases can be defined as interlinked-ambidextrous, showing a benefit in cross-fertilisation of ideas in close relationships<br>Describes that CV units with closer relationships to the core business tend to focus on more strategically aligned projects and hence encounter fewer problems when it comes to commercialising the ideas<br>Interlinked-ambidextrous CV can be found successful as units with the closest relationships to the core business survive in the long term
Taylor & Helfat, 2009	Multiple case study of IBM and NCR	Two cases of technological transitions at IBM and NCR can be defined as interlinked-ambidextrous with different types of organizational linkages involving new core technology and complementary assets across different units<br>Describes three types of organizational linkages for units in charge of the new technology and existing as well as new complementary assets, each requiring ambidextrous management to build new assets, maintain existing ones, and coordinate among them<br>Identifies success factor of influence on organizational linkages contributing to differences in the early success of the technological transitions<br>Interlinked-ambidextrous CV can be found successful in supporting technological transitions by aligning activities involving the new core technology and existing and complementary assets with organizational linkages
Cantarello, Martini, & Nosella, 2012	Single case study of PHYSICA	Case study of a highly innovative technology-based company can be defined as interlinked-ambidextrous with a continuous interplay of exploration and exploitation activities in the front-end innovation process<br>Describes that architectural and contextual issues, as well as managerial responsibility, co-exist and are deeply linked in developing and sustaining ambidexterity.<br>Identifies success factor of both managerial and operational level involvement in a three-phase process to achieve ambidexterity<br>Interlinked-ambidextrous CV can be found successful in supporting both customer's cutting-edge applications and in developing highly innovative solutions by competing in mature and emerging businesses and technologies

Source	Methodology	Category-defining insights
Michl, Gold, & Picot, 2012	Multiple case study of 4 spin-along ventures	CV cases of four spin-along ventures can be defined as interlinked-ambidextrous with deliberate exploring and exploiting to achieve higher innovation performance<br>Describes spin-along as a separate organizational unit that is kept under control and has linkages to the parent firm with the goal of supporting the parent firm's innovation activities as hybrid form of corporate venturing<br>Describes ambidextrous CV with a coordinating and moderating management layer between the corporate parent and the spin-along ventures<br>Identifies success factor of alignment between the spin-along's and parent firm's conflicting goals<br>Interlinked-ambidextrous CV can be found successful in a hybrid form requiring entrepreneurial as well as ambidextrous skills
Leten & Dyck, 2015	Multiple case study of 12 European multinational firms	CV cases of GlaxoSmithKline (GSK), SNCF and Alcatel-Lucent can be defined as interlinked-ambidextrous with prevalent links between CV unit and parent firm<br>Describes that linkages can take various forms, e.g. business unit employees supporting Due Diligence (GSK), business unit executives taking seats in the venturing unit advisory board (SNCF), and co-investments of existing businesses and CV unit (Alcatel-Lucent)<br>Interlinked-ambidextrous CV can be found successful based on separation being combined with mechanisms to link CV unit and parent firm
Weiblen & Chesbrough, 2015	Multiple case study of startup activities in established companies	Outside-in' and 'Inside-out' CV cases such as AT&T, Siemens TBB, SAP Hana and PayPal can be defined as interlinked-ambidextrous with a clear organizational interface to connect external opportunities internally and vice versa<br>Describes alignment with internal business unit through 50% of the staff time from Siemens TBB CV unit (exploiting), while the other 50% is used for the start-ups (exploring)<br>Describes SAP Hana and Paypal to increase the use of their internally developed platforms through start-ups as customers and suppliers<br>Identifies success factor of an organizational interface, ideally with the receiving business unit getting involved before the actual start by choosing the initial topic of a startup pitch session, delegation of some employees to participate in the foundry project, and providing high-level management sponsorship for the project<br>Interlinked-ambidextrous CV can be found successful in helping to hand over a project from startup program to regular business

Source	Methodology	Category-defining insights
Raisch & Tushman, 2016	Longitudinal multiple case study of 6 established companies	CV cases can be defined as interlinked-ambidextrous based on a graduation process with differentiation and integration activities<br>Describes the shift from exploration to exploitation along different phases of the CV process with different differentiation and integration activities across the involved units.<br>Identifies success factors of tight coupling with the corporate level in the exploration phase versus tight coupling with peer units during the exploitation phase<br>Interlinked-ambidextrous CV can be found successful to scale new corporate business transition with shifting relationships along the exploration-exploitation process
Gimmy et al., 2017	Single case study of BMW Garage	CV case of BMW Garage can be defined as interlinked-ambidextrous based on the connection of start-ups with internal business units as (first) clients<br>Describes venture client model in which corporates partner as the first big client with young companies through a 'minimum viable purchase'<br>Identifies success factor of CV unit holding connections to all divisions, including services, IT, manufacturing, and HR, while start-ups stay separated<br>Interlinked-ambidextrous CV can be found successful with the CV unit acting as an intermediary between start-ups and the core business through a defined (interlinking) process
Alänge et al., 2018	Multiple case study of GE FirstBuild, Electrolux Open Innovation, and Lantmännen Greenhouse Accelerator	CV cases of GE, Electrolux and Lantmännen can be defined interlinked-ambidextrous with integration mechanisms built into the operational models<br>Describes integration mechanisms with external networks of innovation partners and propagation of new practices internally<br>Identifies success factors of integration mechanisms to be built into the operational models and upheld by leaders on that level, with support from the top level<br>Interlinked-ambidextrous CV can be found successful as all models are making contributions to the ambidexterity of their parent firms

Source	Methodology	Category-defining insights
Holotiuk & Beimborm, 2018	Single case study of a digital innovation lab from a financial services firm	CV case of the digital innovation lab can be defined as interlinked-ambidextrous with exploitation and exploration becoming more interwoven<br>Describes digital technologies requiring a greater combination of knowledge<br>Identifies problems to incorporate the quickly changing nature of digital technologies which is decreasing the time between implementing an innovation (exploration) and testing and capitalizing it (exploitation)<br>Identifies success factor of integrating exploration and exploitation more densely in a new form of organizational design with adequate methods, lean processes, flexible structures, an innovation-oriented culture, and a proper setup of teams for 'temporal' ambidexterity<br>Interlinked-ambidextrous CV can be found successful for providing new opportunities for firms to realize ambidexterity
Gutmann, Kanbach & Seltman, 2019	Single case study of SAP Industry 4.0 Startup Program	CV case of SAP Industry 4.0 Startup Program can be categorized as interlinked-ambidextrous CV program, as management / unit made sure of selecting external ventures (exploration) based on clear criteria on the internal fit for further exploitation, therefore balancing both within the program<br>Describes specific CV mode of an accelerator program with advantages for corporate business by increasing competitiveness through an extended product ecosystem, cultural change and customer relationship development<br>Exploration through screening of startups with specific criteria especially to establish fit with SAP portfolio, therefore ensuring a future link of the startup solutions with the existing core business<br>Strong support in exploitation in terms of executing the solution together with the SAP product teams and systems, thus also contributing to overall exploitation of SAP's current software portfolio through direct integration support of the startup's solutions<br>Interlinked-ambidextrous CV an be seen as successful, as five startups joined the program after careful selection, resulting in 10 planned integrations into the SAP portfolio, which were still ongoing in the course of the study

Source	Methodology	Category-defining insights
Gutmann et al., 2020	Single case study of Wayra, the corporate accelerator of Telefónica in Germany	CV case of Wayra can be categorized as interlinked-ambidextrous with a program change from an accelerator model to a venture client model, which is more directly linked with the core business<br>Describes matching of startups with specific problems / needs in the core business within selected strategic fields, that way exploring new opportunities to enhance exploitation of core business through the CV unit<br>Exploration outside of the core business with screening startups to participate in an accelerator program, based on specific needs of the core business<br>Exploitation of new opportunities with a paid pilot program taking place with the startup and the core business, especially also supporting the exploitation of the existing core business with new innovative startup solutions<br>Interlinked-ambidextrous setup can be seen as a more successful approach in contrast to former separated-integrated setup, in which startups were less collaborating with the core business. However, some pain points still exist from the startup point of view, i.e. in terms of decision-making and speed of collaboration
Shin & Cho, 2020	Single case study of Samsung Creative-Lab and multiple case study of its ventures	CV case of Samsung Creative-Lab can be categorized as contextual-ambidextrous due to enablement of all employees, especially the R&D workforce, to identify and commercialize ideas in addition to their operational dayjob<br>Describes inside-out system where employee ideas can be spun-off for commercialization<br>Identifies relevant contribution of the CV activity for corporate leadership, culture, new ways of working and overall entrepreneurial orientation<br>Contextual-ambidextrous CV can be found successful with participation reaching 1% of the R&D workforce 3 years earlier than planned, and having 45 corporate ventures spun-off whose value tripled from the time of the spin-off, including ventures such as Linkflow with more than $50m in value, and mangoslab with more than $7m of revenues in the first year (overall contribution to core organization remains to be seen)
